# Hepatic transcriptomic analysis reveals differential regulation of metabolic and immune pathways in three strains of chickens with distinct growth rates exposed to mixed parasite infections

**DOI:** 10.1186/s13567-024-01378-8

**Published:** 2024-09-28

**Authors:** Oyekunle John Oladosu, Henry Reyer, Rosemarie Weikard, Beatrice Grafl, Dieter Liebhart, Cornelia C. Metges, Christa Kühn, Gürbüz Daş

**Affiliations:** 1 Research Institute for Farm Animal Biology (FBN), Wilhelm-Stahl-Allee 2, 18196 Dummerstorf, Germany; 2 Clinic for Poultry and Fish Medicine, University of Veterinary Medicine Vienna, Veterinärplatz 1, 1210 Vienna, Austria; 3 Friedrich-Loeffler-Institute, Federal Research Institute for Animal Health, Südufer 10, 17493 Greifswald-Insel Riems, Germany; 4 Agricultural and Environmental Faculty, University Rostock, Justus-Von-Liebig-Weg 6, 18059 Rostock, Germany

**Keywords:** *Ascaridia galli*, Growth performance, *Heterakis gallinarum*, *Histomonas meleagridis*, immune response, metabolic response, resource allocation, trade-off

## Abstract

During parasite infections, the liver may prioritise immune-related pathways over its metabolic functions. Intestinal infections caused by *Ascaridia galli* and *Heterakis gallinarum* impair feed intake, nutrient absorption, and weight gain. *Histomonas meleagridis,* vectored by *H. gallinarum*, can also damage liver tissues, potentially impairing liver functions. This study examined the hepatic gene expression in three strains of chickens: Ross-308 (R), Lohmann Brown Plus (LB), and Lohmann Dual (LD), 2 weeks after an experimental infection (*n* = 18) with both *A. galli* and *H. gallinarum* or kept as uninfected control (*n* = 12). Furthermore, *H. gallinarum* infection led to a co-infection with *H. meleagridis*. The mixed infections reduced feed intake and the average daily weight gain (*P* < 0.001). The infections also increased the plasma concentrations of alpha (1)-acid glycoprotein and the antibody titre against *H. meleagridis* (*P* = 0.049), with no strain differences (*P* > 0.05). For host molecular response, 1887 genes were differentially expressed in LD, while 275 and 25 genes were differentially expressed in R and LB, respectively. The up-regulated genes in R and LD were mostly related to inflammatory and adaptive immune responses, while down-regulated genes in LD were involved in metabolic pathways, including gluconeogenesis. Despite performance differences among the strains, worm burdens were similar, but hepatic molecular responses differed significantly. Moreover, there was an indication of a shift in hepatic functions towards immune-related pathways. We, therefore, conclude that the liver shifts its functions from metabolic to immune-related activities in chickens when challenged with mixed parasite species.

**Supplementary Information:**

The online version contains supplementary material available at 10.1186/s13567-024-01378-8.

## Introduction

The efforts made for more than half a century to genetically select broilers have led to an increase in growth performance of over 400% [1]. However, this achievement has potentially brought about unintended consequences, such as compromising broilers’ capacity to effectively handle physiological, behavioural, and immunological challenges [2]. For example, a meta-analysis investigating the trade-off between growth and immune function in poultry corroborated that genetic selection for rapid growth compromises immune function [3]. The diminished capacity of broilers to contend with the challenges that trigger immune responses may be linked to allocating finite resources between physiological processes [4, 5]. Resource allocation, determined by an organism’s genetic disposition and immediate physiological needs, often occurs through the prioritised distribution of nutrient resources, which can create an imbalance in the allocation of resources between different physiological processes [6]. Allotting nutrient resources to various physiological processes appears to be a fundamental evolutionary mechanism crucial for an organism’s survival [7].

There have already been reported cases of broiler chickens showing poor tolerance to challenges, particularly concerning infections. For example, fast-growing broilers exhibited greater susceptibility to Marek’s disease than their slow-growing counterparts [8]. Additionally, different susceptibilities to bacterial infections have been demonstrated among chicken strains selected for digestive efficiencies [9] and performance traits [10]. An examination of laying hens exposed to mixed parasite infections in chickens [11] revealed a potential trade-off between defence and performance traits. Our previous study found that birds with high-performance levels had a reduced laying capacity within 2 weeks of exposure to nematode infections compared to a low-performing strain. This timeframe also represents the period when a prompt immune response is initiated against these infections [11, 12]. Further comparison of how well three strains of chickens tolerate mixed nematode infections revealed that the Ross 308 strain, despite having superior growth performance, was the least tolerant compared to a strain with intermediate performance and another with low performance. The latter two strains are presumably not under selective pressure for fast growth, unlike the Ross 308 strain [13].

Helminth infections are becoming increasingly prevalent, particularly in regions that have prohibited cage-housing systems primarily for welfare reasons [14–16]. The most frequently reported infections in chickens are caused by *Ascaridia galli* and *Heterakis gallinarum.* These infections have been associated with impaired host performance, specifically through reduced feed intake and nutrient utilisation efficiency [17–19]. Infections with ascarids are renowned for inducing a localised immune response, primarily in the intestine [20–22]. Furthermore, these infections often coincide with the protozoon *Histomonas meleagridis*, which is known to cause liver damage and trigger further immuno-pathophysiological responses [23–25]. However, the molecular responses to these infections, particularly in terms of the underlying immuno-pathophysiological mechanisms, remain poorly understood and warrant further investigation. Under natural conditions, chickens are infected with multiple species of helminths [26], potentially including protozoa, which suggests that the impact of multiple-species infections is of greater practical relevance than those of single-species infections.

The chickens’ immune system employs different immune responses, such as T-helper (Th)-1 and Th2 pathways, to address intracellular (e.g., viruses) and extracellular (helminths) pathogens, respectively [27]. During viral infection of chickens, it was observed that an increase in the Th1 cytokine IFN-γ was linked to reduced expression of the Th2 marker interleukin-13 (IL-13) in both ileal tissue and the spleen. Conversely, in chickens infected with *A. galli*, expression of IL-13 mRNA was observed in both the spleen and ileum on day 14 post-infection, together with diminished IFN-γ expression [27]. Such an outcome implies a polarisation of host immune responses to intra and extracellular pathogens in the same host. However, infection with *H. meleagridis* has been associated with increased production of IFN-γ [28]. Hence, the co-infection of ascarids and histomonas in chickens could be an intriguing model for understanding the relationship between the two parasite species, which may induce different immune responses [27, 28].

Moreover, due to the liver’s central role in nutrient metabolism and detoxification [29], simultaneous infection with *H. meleagridis* during a nematode infection can further exacerbate the trade-off between performance and immune defence by prioritising immune functions over its metabolic functions. This exacerbation is particularly prevalent in high-performing strains. Therefore, we have formulated a hypothesis suggesting a possible switch in the hepatic functions from metabolic to immune-related functions in chickens infected with mixed parasite species. This shift may correlate with the host animal’s performance level, for example, their growth rate. The objective of this study was to identify specific hepatic genes and pathways that contribute to the differences in host responses to mixed nematode infection with concurrent histomonosis in three strains of chickens with distinct differences in growth rate.

## Materials and methods

### Samples and design

A cohort of 30 male chicks representing three distinct chicken strains developed for specific purposes—namely egg production (Lohmann Brown Plus, LB), meat production (Ross-308, R), or a dual-purpose combination (Lohmann Dual, LD)—were used for this study. The R and LB strains were chosen to evaluate growth performance as they were the most divergent. In contrast, LD birds—which exhibit a growth rate between the two extremes—were included as intermediary strains. This cohort of birds originated from a previous study [13], where the resistance and tolerance of birds with different growth rates exposed to mixed nematode infections were investigated for 9 weeks post-infection (wpi).

For the present study, only the birds necropsied at 2 wpi were investigated and are thus described here. The investigation involved the experimental induction of infections in a subset of birds (*n* = 18, i.e. 6 per strain) at the age of 1 week, with a total of 500 infective eggs of both *A. galli* and *H. gallinarum*, thereby inducing mixed ascarid infections. Concurrently, another subset of birds was maintained as the uninfected controls (*n* = 12, i.e. 4 per strain). The birds were randomly allocated to pens, each containing birds of the same strain. The infected and uninfected pens were in two separate rooms within the same experimental stable. All birds were uniquely identified with wing tags on the day of infection to ensure the collection of individualised samples. Birds were transferred to individual cages one day before the necropsy to ensure the collection of individual total daily faecal samples (24 h faeces). Necropsies were performed on infected and uninfected birds from each strain (*n* = 10 per strain) to assess the presence and intensity of nematode infections. Uninfected control birds were investigated for accidental infections to preclude any potential confounding influences. Pen-based feed consumption and average body weight were documented before infection at 0, 1, and 2 wpi.

During the necropsy at 2 wpi, faecal, blood, and liver specimens were obtained and promptly frozen and stored at −80 °C for subsequent analyses. The study adhered to ethical standards governing animal welfare, encompassing protocols for animal care, handling, stunning, and necropsies. Additionally, it received approval from the State Ethics Committee for Animal Experimentation (Mecklenburg-Western Pomerania State Office for Agriculture, Food Safety, and Fishery, Germany; permission no: AZ: 7221.3-1-066/15). The experimental infection procedures adhered to the guidelines outlined by the World Association for the Advancement of Veterinary Parasitology for poultry [30].

### Bird management

The birds were raised using a floor husbandry system with wood shavings as litter material. The litter remained unchanged during the experimental period and was adjusted for total body weight per m^2^ to maintain consistent litter conditions for all strains across different pens. An automated system was used to regulate climatic conditions such as temperature, lighting, and aeration to maintain consistency within and between rooms. Both feed and water were provided ad libitum, with all strains receiving the same commercial diet (12.6 MJ of metabolisable energy and 219 g of crude protein per kg of feed). No vaccinations or medical interventions, including anthelmintics or antiprotozoal drugs, were administered to the birds before or after infection. Pen-based data was used to calculate the average daily feed intake, daily weight gain, and feed conversion ratio during the infection period, which lasted from the day of infection to 14 days post-infection.

### Infection procedure

The infection material originated from the intestines of naturally infected free-range chickens collected in various slaughterhouses and farms in northern Germany. The preparation methods and incubation conditions for the infection material were detailed by Stehr et al. [12]. On the day of infection (7 days of age), *A. galli* and *H. gallinarum* incubation media were filtered through a sieve (36 µm mesh size). This process was followed by rinsing to gather the washed eggs in a saline solution (NaCl, 0.9%). Using morphological classification criteria [31], only fully embryonated eggs considered infectious were quantified to establish the count and percentage of embryonated eggs per mL of suspension. The single infection dose for each worm species was adjusted at 250 embryonated eggs per 0.1 mL of NaCl (0.9%). The adjusted dose was orally administered to each bird in a final inoculum of 0.2 mL of NaCl to obtain a total of 500 eggs with equal proportions of the two worm species. Oral administration of the infective dose utilised a 5 cm oesophageal cannula, while the uninfected control birds received an oral sham treatment with an equivalent volume (0.2 mL) of NaCl solution.

### Worm burden determination

At the end of 2 wpi, the birds (aged 3 weeks) were killed by stunning using a bolt shoot followed by bleed. Before necropsy, a standardised partial emptying of the intestine was facilitated by subjecting the birds to a 3 h fasting period. During post-mortem procedures, the gastrointestinal tract was extracted, and the caecum and small intestine (SI) were isolated. The SI was divided into the jejunum and ileum at the Meckel’s diverticulum. The duodenum was omitted from quantification due to macroscopic examinations indicating that this intestinal section is not the typical habitat for *A. galli*. The jejunum and ileum were opened longitudinally, and the intestinal contents were separated by section and subsequently washed individually through sieves with a mesh size of 36 µm. The quantification of tissue-dwelling *A. galli* larvae was done through digestion procedures described earlier [12], with special attention given to the jejunal section, which is considered the favoured site for larval stages [32].

*Heterakis gallinarum* worms were isolated from the luminal contents by rinsing the opened caeca in sieves (mesh sizes 20–36 µm). Both *A. galli* and *H. gallinarum* worms, sourced from individual birds, were then individually placed in Petri dishes for counting. Uninfected birds were examined for worm presence in the SI (tissue and lumen) and caecum to exclude accidental infections with either nematode. The total worm burden for each species included all lumen and tissue worms.

### Quantification of antibodies against ascarids and *Histomonas meleagridis*

Using EDTA tubes, blood samples were collected from the birds at slaughter and immediately placed on ice. Centrifugation at 2500 × *g* for 20 min at 4 °C was performed to separate plasmas, which were stored at −20 °C until further analysis.

Anti-ascarid-specific IgY levels in EDTA plasma samples were quantified using an ascarid-specific enzyme-linked immunosorbent assay (ELISA) as described previously [33]. The laboratory-specific intra-assay coefficient of variability (CV) and inter-assay CV for this analysis were determined to be 5.0% and 8.4%, respectively. The same set of plasma samples (*n* = 30) were examined to detect *H. meleagridis*-specific antibodies using an ELISA protocol [34].

In summary, ELISA plates were coated with rabbit anti-Histomonas serum [35] at a 1:10 000 dilution in carbonate buffer. Following overnight incubation at 4 °C and subsequent washing with PBS-Tween 20 (0.05% PBST), the plates underwent treatment with blocking buffer (Starting Block™T20 PBS, Pierce Biotechnology, Rockford, USA). The suitably diluted *H. meleagridis* antigen was introduced to each well and incubated for 1 h at room temperature, followed by another washing step. The plasma samples were diluted at 1:500 with a blocking buffer and incubated for 1 h at room temperature. Each plate included positive and negative control sera obtained from chickens experimentally infected with *H. meleagridis*. After washing, goat anti-chicken IgG-horseradish peroxidase (Southern Biotech, Birmingham, AL, USA) was added for 1 h before another washing step, and tetramethylbenzidine substrate solution (TMB; Calbiochem, Merck, Vienna, Austria) was employed for 15 min in the dark. Optical density measurements were taken at a wavelength of 450 nm. The cut-off value of 0.54 nm was applied to differentiate positive and negative results.

### Measurements of plasma metabolites and acute-phase protein

The levels of glucose, triglycerides (TG), and total cholesterol were measured using plasma samples collected with fluoride-EDTA. In addition, EDTA plasma samples were used to measure total protein, albumin, lactic acid, uric acid (UA), low-density lipoprotein (LDL), and high-density lipoprotein (HDL) levels. Globulin was calculated as the difference in total protein and albumin concentration. The measurement of plasma metabolite concentrations was carried out using an automatic enzymatic analyser (ABX Pentra 400, HORIBA Medical, Montpellier, France). Commercial kits were employed for individual metabolite assays, including glucose (Kit No. A11A01667, Axon Lab AG, Reichenbach/Stuttgart, Germany), TG (Kit No. A11A01640, Axon Lab AG), UA (Kit No. A11A01670, Axon Lab AG, Reichenbach/Stuttgart, Germany), lactic acid (Kit No. A11A01721, Axon Lab AG), total cholesterol (Kit No. 553-300, MTI Diagnostics, Idstein, Germany), total protein (Kit No. 553-412, MTI Diagnostics, Idstein, Germany), albumin (Kit No. A11A01664, Axon Lab AG, Reichenbach/Stuttgart, Germany), LDL cholesterol (Kit No. A11A01638, Axon Lab AG, Reichenbach/Stuttgart, Germany), and HDL cholesterol (Kit No. A11A01636, Axon Lab AG, Reichenbach/Stuttgart, Germany). The acute-phase protein, alpha (1)-acid glycoprotein (AGP), was measured in plasma samples using a commercial ELISA kit following the manufacturer’s instruction (Life Diagnostics, West Chester, USA, Catalogue number: AGP-5).

### Quantification of *Histomonas meleagridis* DNA in host faeces

A real-time polymerase chain reaction (PCR) targeting the 18S rDNA of the parasite was used to detect *H. meleagridis* DNA directly in the host faeces [36]. Protozoan quantification was achieved using a standard curve of Ct values generated from DNA samples of *H. meleagridis,* which were obtained from defined quantities of in vitro cultivated flagellates. To establish the standard curve, serial dilutions of cultured *H. meleagridis*, spanning from 10^6^ to −10^–1^ protozoa/mL, were prepared. These dilutions were stored at −20 °C before being thawed and subjected to DNA extraction using the DNeasy® Blood & Tissue Kit (Qiagen, Vienna, Austria) per the manufacturer’s protocol.

Faecal samples (*n* = 30) were collected from individual birds across all groups in the infection experiment and then thawed. Each sample, weighing 200 mg, was homogenised using the Qiagen TissueLyser (Qiagen). Subsequently, DNA was extracted from individual faecal samples using the QIAamp® Fast DNA Stool Mini Kit (Qiagen), following the manufacturer’s recommendations. The extracted DNA was used for real-time PCR conducted in a 20 μL reaction mixture on the Agilent Mx3000P (Agilent Technologies, Santa Clara, CA, USA) using TaqMan chemistry, Brilliant III UltraFast QPCR Master Mix (Agilent Technologies, Santa Clara, CA, USA) with 30 nM ROX as a reference dye, 0.2 μM primers, and 0.3 μM TaqMan probe. The thermal profile of reactions comprised an initial 15 min denaturation at 95 °C, followed by 40 cycles of 15 s at 95 °C and 30 s at 60 °C. Fluorescence was detected and recorded at each cycle during the 60 °C step.

### RNA extraction library construction and RNA sequencing

Total RNA was isolated from the 30 liver samples. After grinding the frozen tissue under liquid nitrogen, 30 mg of the samples were used for RNA extraction using TRIzol (Invitrogen, Darmstadt, Germany) according to the manufacturer’s protocol. The RNA was precipitated and subsequently purified using the NucleoSpin RNA II kit (Macherey & Nagel, Düren, Germany), which utilises a column-based technique and includes DNase digestion. PCR verified the absence of genomic DNA in the RNA samples with primers specific for avian *PPARGC1A*. Analysis of RNA integrity on the 2100 Bioanalyzer instrument (Agilent Technologies, Germany) resulted in RNA integrity numbers (RIN) of 9.0 ± 0.3. Based on the total RNA from the liver, sequencing libraries with a specific index for each sample were generated using the Truseq RNA sample prep kit (Illumina, San Diego, USA). The libraries were checked on the 2100 Bioanalyzer instrument and then sequenced using the HiSeq 2500 Sequencing System (Illumina) to obtain paired-end sequences (2 × 100 bp). Initial checking and trimming of raw sequencing data was done using FastQC software [37], Cutadapt v. 1.16 [38] and Quality Trim v. 1.6.0 [39]. Clean reads were mapped to the *Gallus gallus* reference genome (Gga5, annotation Ensembl release 92) with HISAT2 v.2.1.0 [40] and read counts for each feature were obtained with featureCounts (subread v.1.6.2) [41].

### Differential expression analysis and enrichment analysis

Differential expression and principal component analyses (PCA) were performed using DESeq2 in the R programming language. The comparisons performed include (i) the effects of mixed infections within each strain and (ii) the strain differences within the infection group. The p-values were corrected using the Benjamini–Hochberg procedure to control the false discovery rate (FDR). Transcripts with an adjusted *P*-value < 0.05 were considered differentially expressed genes (DEGs). The Database for Annotation, Visualisation and Integrated Discovery (DAVID) was used to assess the enrichment of DEGs in biological pathways employing the Gene Ontology (GO) database and Kyoto Encyclopedia of Genes and Genomes (KEGG) database. In addition, genes annotated to terms of Th1 (GO:0042088), Th2 (GO:0042092), Th17 (GO:0072538), and Treg (GO:0045580) in the EMBL-EBI GO database [42] were used to investigate the enrichment of DEGs in immune processes.

### Statistical analyses of phenotypic data

The statistical model for average daily feed intake (AFI), average daily weight gain (ADG), feed conversion ratio (FCR), histomonads excretion in 24 h faeces (histomonads per day, i.e. HpD), anti-ascarid IgY, anti-histomonas antibody titre, and AGP concentration included the fixed effects of infection, host strain, and their interaction. The analysis of worm burden data excluded control birds that were free of nematodes, with the host strain being the only fixed effect in the model. All variables were subjected to ANOVA using the PROC GLM procedure of the SAS On Demand for Academics cloud-based software (2021 SAS Institute Inc., Cary, NC, USA). Before conducting statistical analysis, the data for HpD, AGP, anti-ascarid IgY, and anti-histomonas antibody underwent a log transformation [Ln(y + 1)] to address the heterogeneity of variance and ensure an approximately normal distribution of the data.

## Results

### Host performance and infection phenotypes

**Figure 1:**
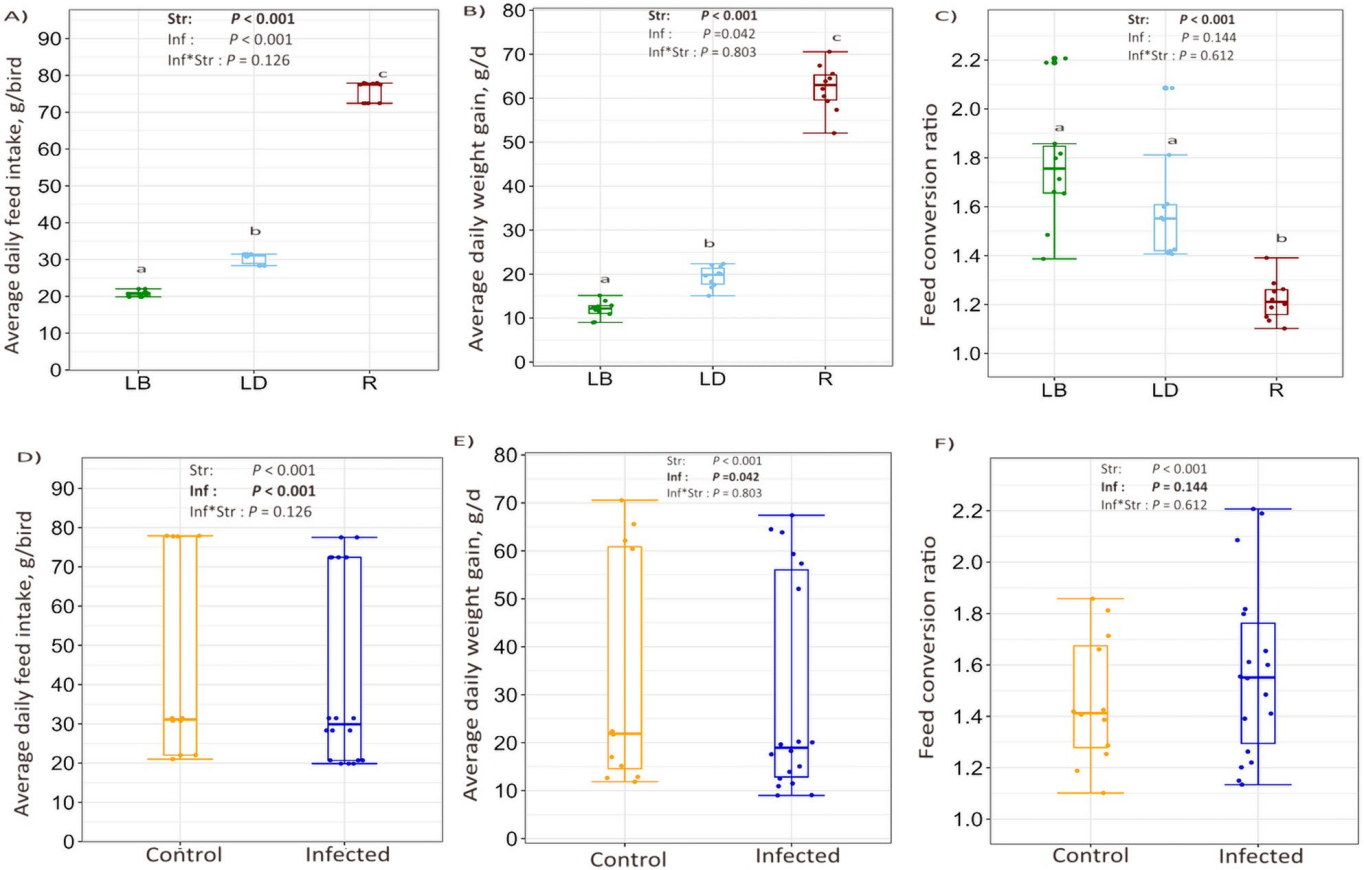
**Growth performance, feed intake, and feed conversion ratio in three chicken strains with distinct growth rates following mixed-parasite infections.** The fixed effects of host strain, (**A**–**C**) infection (**D**–**F**), and their interaction (not shown) on average daily feed intake (g/bird), average daily weight gain (g/d), and feed conversion ratio within the three strains of birds, namely, Lohmann Brown (LB), Lohmann Dual (LD), and Ross (R). Each dot represents the average value for each variable, with bars depicting the standard errors of the mean. The line inside the boxplots shows the sample median, while the lower and upper end of the box represent the 25th and 75th quantiles, respectively (*n* per strain = 10, *n* infected birds = 18, *n* birds in control = 12).

All three strains differed (*P* < 0.001) in their growth performance (Figure 1A–C). The AFI and ADG were highest in R and lowest in LB strain (*P* < 0.001). The LB and R strains had the highest and the lowest FCR, respectively, while no significant difference was quantified between the LB and LD strains (*P* = 0.144). The mixed infections reduced AFI (*P* < 0.001), which consequently impaired ADG (*P* = 0.042). Despite causing a numerical increase, the infections did not influence FCR (*P* = 0.144) (Figure 1D–F). Moreover, there was no significant interaction effect due to strain × infections (*P* > 0.05) for the performance data presented (interaction data not shown).

**Figure 2:**
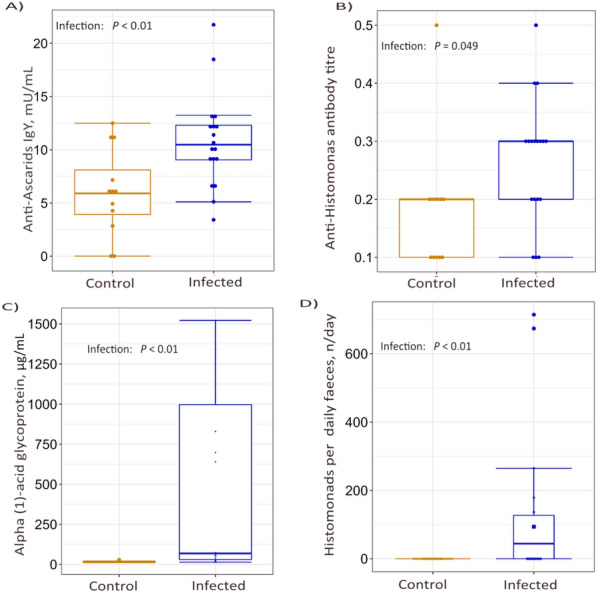
**Infection proxies.** Plasma antibody titres against **A** Ascarids, and **B** *Histomonas meleagridis*, as well as **C** the plasma concentration of the acute-phase protein AGP, and **D** the PCR quantification of histomonads per total daily faeces (HPD) were measured. Each dot in the boxplot represents an individual bird, with uninfected birds represented by orange dots and infected birds represented by blue dots. The lower and upper ends of the box represent the 25th and 75th quantiles, respectively. Statistical analyses are based on log-transformed data [log(y + 1)], while visualisation is based on untransformed data. (*n* infected birds = 18, *n* birds in control = 12).

Data for worm burden with *A. galli* and *H. gallinarum* in the experimentally infected birds are presented in Table 1. No significant differences (*P* > 0.05) were observed in the total average worm burden of both *A. galli* and *H. gallinarum* between the three strains. The birds kept as uninfected controls had no mature worms or larvae. Similarly, the PCR quantification of histomonads per gram of faeces (HpG) and in total daily faeces (HpD), anti-histomonas antibody titre and alpha (1)-acid glycoprotein (AGP) concentrations were not significantly different across strains (*P* > 0.05). However, both LB and LD produced higher anti-ascarid IgY (*P* < 0.05) than the R strain (Table 1). Compared to the controls, the infected birds had significantly higher HpD, AGP, and anti-ascarid IgY levels (*P* < 0.05). The anti-histomonas antibody titre was significantly higher in infected birds (*P* = 0.049) (Figure 2). In addition, one Ross and two LD birds had macroscopic lesions on the liver, characteristic of histomonas infections.

**Table 1:** **Effects of host genotype on the mixed-parasite infection parameters**

Variable	n	LB	LD	R	SE	P
*A. galli*, n/bird	18	105	101	140	26.77	0.544
*H. gallinarum*, n/bird	18	28	24	18	11.63	0.850
Anti-ascarid IgY, mU/mL	30	4.29^a^	5.82^a^	2.53^b^	0.83	0.006
Anti-*H. meleagridis* titre	30	0.22	0.24	0.22	0.04	0.890
HpG	30	0.67	23.13	4.69	8.51	0.235
HpD	30	8.23	408.23	411.79	176.86	0.098
AGP µg/mL	30	293	271	215	147.00	0.925

Data are presented as the LS-means and their standard errors (SE) for each strain of chickens. Uninfected control chickens were excluded for *A. galli* and *H. gallinarum* burdens.

*LB* Lohmann Brown, *LD* Lohmann Dual Plus, *R* Ross 308, *HpG* number of histomonads per gram faeces, *HpD* number of histomonads excreted within 24 h, *AGP* alpha (1)-acid glycoprotein.

Means with different superscripts are significantly different (*P* < 0.05). There were no significant interaction effects between genotype and infection on anti-ascarid IgY, anti-histomonas titre, HpG, HpD and AGP (*P* > 0.05).

Ross birds showed significantly higher HDL cholesterol concentrations than LD and LB strains (Table 2), while LD birds had lower LDL cholesterol levels than LB and Ross birds. Additionally, infections considerably increased plasma concentrations of total protein and globulin (*P* < 0.05) and decreased total cholesterol levels (*P* = 0.026). The model indicated a significant interaction between strain effect and infection effect for total cholesterol (*P* = 0.047), the difference being found in the LB strains, where cholesterol levels were lower in infected LB birds than in controls (*P* = 0.037). The plasma concentrations of uric acid, lactic acid, glucose, triglycerides, and albumin remained unchanged by infections, host strain, or interaction effects (*P* > 0.05).

**Table 2:** **Effects of strain and the mixed-parasite infection on the concentration of plasma metabolites in chicken**

Variable	n	Strain effect					Infection effect				Interaction, *P* ≤
		LB	LD	Ross	SE	P ≤	Control	Infected	SE	*P* ≤	Gen * Inf
Glucose, mmol/L	28	13.55	13.23	13.17	0.48	0.825	13.19	13.43	0.40	0.650	0.076
Chol, mmol/L	29	3.59	3.32	3.87	0.17	0.100	3.83	3.35	0.15	0.026	0.047
TG, mmol/L	29	0.41	0.37	0.38	0.02	0.380	0.37	0.41	0.02	0.127	0.118
LA, mmol/L	29	6.07	5.89	5.79	0.34	0.859	5.58	6.25	0.33	0.117	0.082
LDL, mmol/L	30	0.76^a^	0.57^b^	0.86^a^	0.06	0.011	0.79	0.67	0.05	0.100	0.238
HDL, mmol/L	27	1.79^a^	1.93^a^	2.31^b^	0.11	0.006	2.05	1.97	0.09	0.556	0.366
Uric Acid, µmol/L	29	364.56	316.93	417.58	63.29	0.571	414.93	317.78	59.67	0.211	0.113
Albumin, g/L	30	12.81	13.33	13.30	0.49	0.706	13.05	13.24	0.44	0.736	0.127
Protein, g/L	30	30.23	31.76	31.62	1.79	0.803	27.97	34.44	1.60	0.005	0.585
Globulin, g/L	30	17.43	18.43	18.31	1.41	0.859	14.91	21.19	1.26	0.001	0.814

Chol: total cholesterol, TG: triglyceride, LA: lactic acid, LDL: low-density lipoprotein cholesterol, HDL: high-density lipoprotein cholesterol. Values with different superscript are significantly different (*P* < 0.05).

### RNA sequencing and differential gene expression analysis

A total of 15 035 expressed transcripts were obtained in the liver of sampled chickens by RNA-Seq expression profiling. Of these, 10 626 were annotated with a known gene symbol; hence, only genes with a known gene symbol were included in the gene set enrichment analyses.

**Figure 3:**
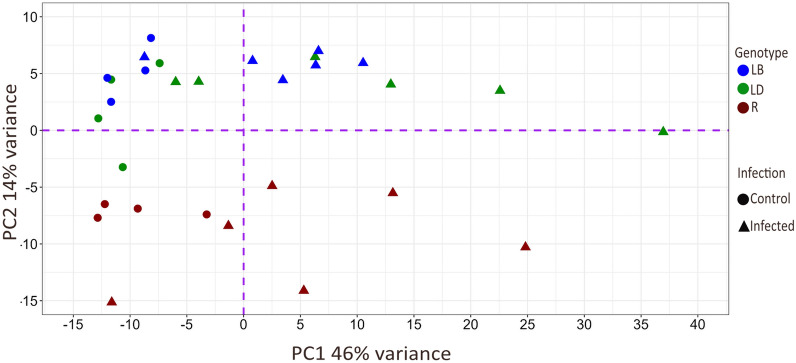
**Principal component analysis showing the overall separation of host-strains and infection groups based on the RNA-sequencing profiles.** Principal component analysis (PCA) was performed on gene expression data to visualise the clustering of samples based on infection status and genotype. The plot displays two principal components, PC1 and PC2, which account for 46% and 14%, respectively, of the total variance in the data. Each point represents an individual sample, shape-coded according to infection group (Infected and Control) and coloured by strain (LB, LD, R).

As shown in Figure 3, the PCA indicated a distinct separation of infected birds from their uninfected counterparts in terms of their overall gene expression patterns across all three strains (i.e. see the dispersion on the PC1 axis with 46% of variation explained). Similarly, R birds separated considerably from LB and LD birds (PC2 axis, 14% of variation). In contrast, no separation of the two latter strains was observed in their overall gene expression patterns.

### Liver transcriptome response to mixed parasite infection in three strains

**Figure 4:**
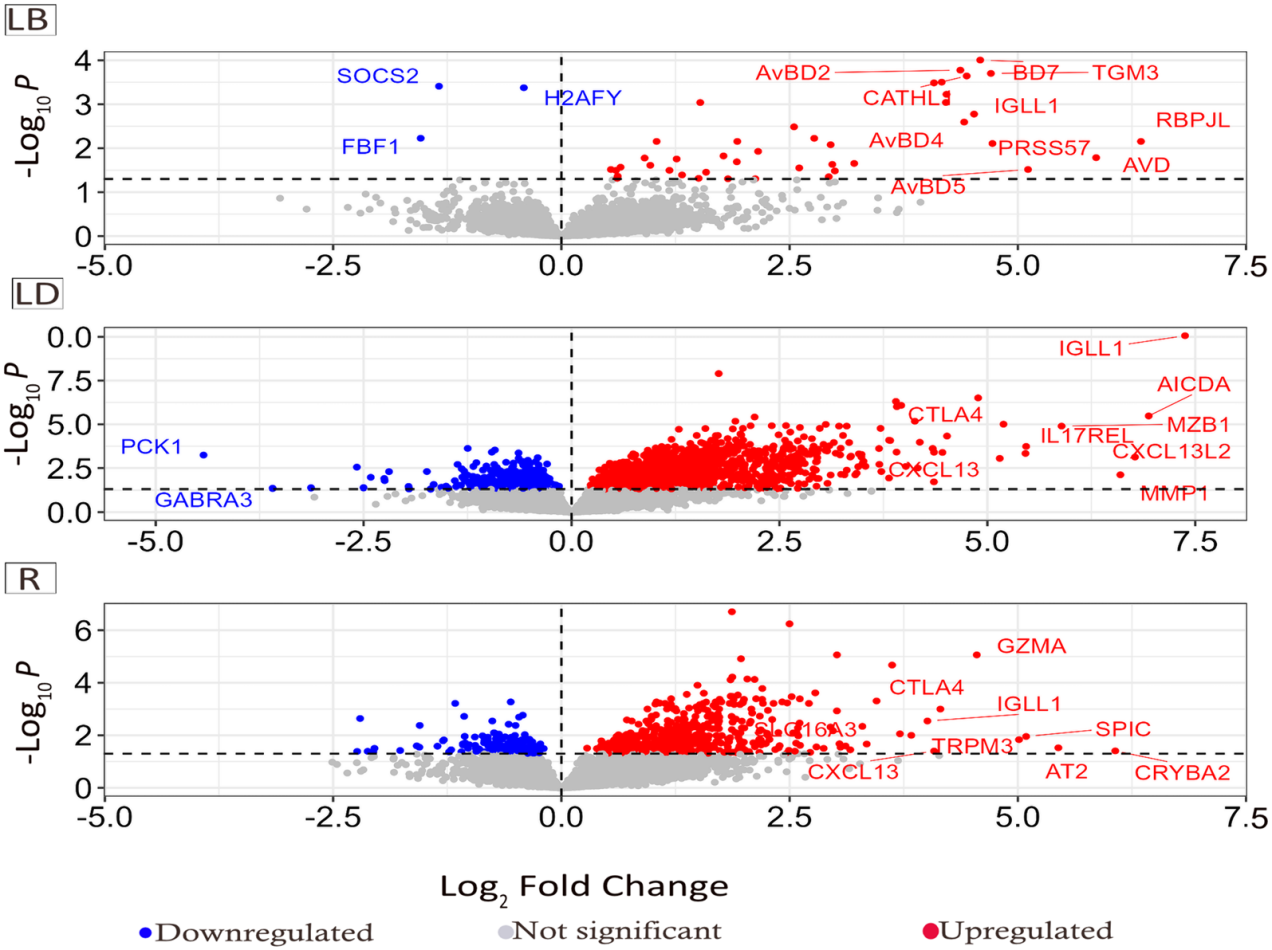
**Volcano plots showing differentially up-regulated and downregulated genes due to infection and strain effects.** Volcano plots showing differentially up-regulated genes (red dots) and downregulated genes (blue dots) due to infection effects (infected versus non-infected, FDR < 0.05) in **A** Lohmann Brown, **B** Lohmann Dual, and **C** Ross strains. Non-significant genes are shown in grey dots.

When comparing the infection effect (i.e. infected versus control), the abundance of 74 genes (69 up-regulated, 5 down-regulated) was significantly altered in the LB strain (Figure 4). *Suppressor of cytokine signalling 2* (*SOCS2*, log_2_FC = −1.34, FDR = 0.0004), *MacroH2A.1 histone* (*H2AFY*, log_2_FC = −0.41, FDR = 0.0004) and *Fas binding factor 1* (*FBF1*, log_2_FC = −1.54, FDR = 0.006) were among the significantly down-regulated DEGs in LB chickens, while *recombination signal binding protein for immunoglobulin kappa J region like (RBPJL*, log_2_FC = + 6.35, FDR = 0.007), *Avidin* (*AVD*, log_2_FC = + 5.86, FDR = 0.016) and *Avian beta-defensin 5* (*AvBD5*, log_2_FC = + 5.11, FDR = 0.030) were among the most strongly up-regulated genes (Figure 4A).

The effects of infection on the LD strain revealed 1761 DEGs that were significantly up-regulated and 779 DEGs that were significantly down-regulated (FDR < 0.05) (Figure 4). *Phosphoenolpyruvate carboxykinase (PCK1*, log_2_FC = −4.42, FDR < 0.001), *Gamma-aminobutyric acid type A receptor alpha 3* (*GABRA3*, log_2_FC = −3.59, FDR = 0.004), and *NK6 homeobox 3* (*NKX6-3*, log_2_FC = −3.13, FDR = 0.004) were the most down-regulated. Immunoglobulin *lambda-like polypeptide* (*IGLL1*, log_2_FC = + 7.38, FDR < 0.0001), *Avidin* (*AVD*, log_2_FC = + 7.68, FDR < 0.001) and *Activation-induced cytidine deaminase* (*AICDA*, log_2_FC = + 6.94, FDR < 0.001) were the most up-regulated in LD birds.

A lower number of infection-induced DEGs (*n* = 904) were identified in the R strain compared to the LD strain, but these were nevertheless higher than the DEGs expressed in LB birds. In Ross, 724 DEGs were significantly up-regulated, and 180 DEGs were down-regulated (Figure 4). Among the most up-regulated DEGs in the R strain were *Crystallin beta A2* (*CRYBA2*, log_2_FC = + 6.07, FDR = 0.039), *ADP-ribosyltransferase 1E* (*AT2*, log_2_FC =  + 5.44, FDR = 0.029), and *Spi-C transcription factor* (*SPIC*, log_2_FC = + 5.09, FDR = 0.011), while *ATPase plasma membrane Ca2*+ *transporting 2* (*ATP2B2*, log_2_FC = −2.20, FDR = 0.002), *RALY heterogeneous nuclear ribonucleoprotein* (*RALY*, log_2_FC = −2.24, FDR = 0.04)*,* and *growth hormone-releasing hormone receptor* (*GHRHR,* log_2_FC = −2.06, FDR = 0.042) were the top three most down-regulated DEG in R strain.

**Figure 5:**
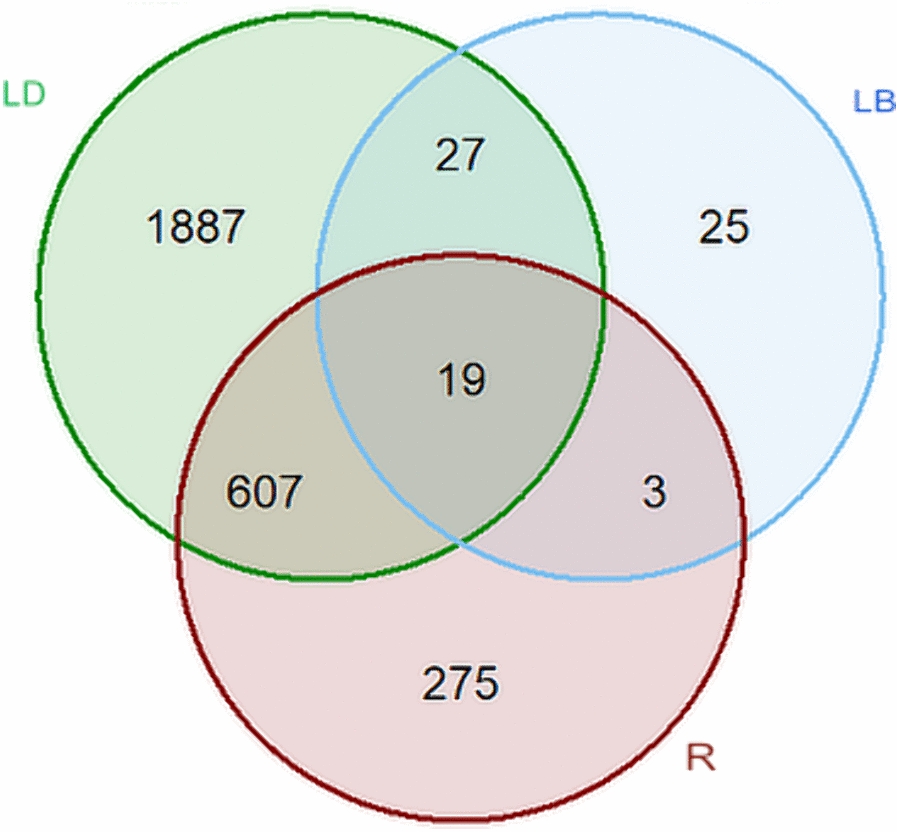
**Pairwise comparison of DEGs due to the infection effect within strains.** Venn diagram showing the number of overlapping and unique differentially expressed genes due to infection effects (infection versus contrast) in all 3 strains of birds.

The number of unique DEGs within each strain and the overlapping DEGs in the three strains after exposure to infection (i.e. infection effects in LB, LD, and R strains) are shown in Figure 5. Up to 62% of the DEGs in LB birds were also considered DEGs in the LD strain (*n* = 46). Between the R and LD strains, 626 DEGs were shared, while only 22 DEGs were common between R and LB. A total of 19 genes, including *Ral guanine nucleotide dissociation stimulator like 1* (*RGL1*), *Complement C7* (*C7*), *Superoxide dismutase 3*, *extracellular* (*SOD3*), *immunoglobulin lambda-like polypeptide 1* (*IGLL1*), *chitinase acidic* (*CHIA*), *member RAS oncogene family* (*RAB37*), and *fatty acid-binding protein 3* (*FABP3*) were significantly more highly expressed in infected chickens of all three strains (Additional file 1).

#### Pairwise comparison of DEGs between strains

**Figure 6:**
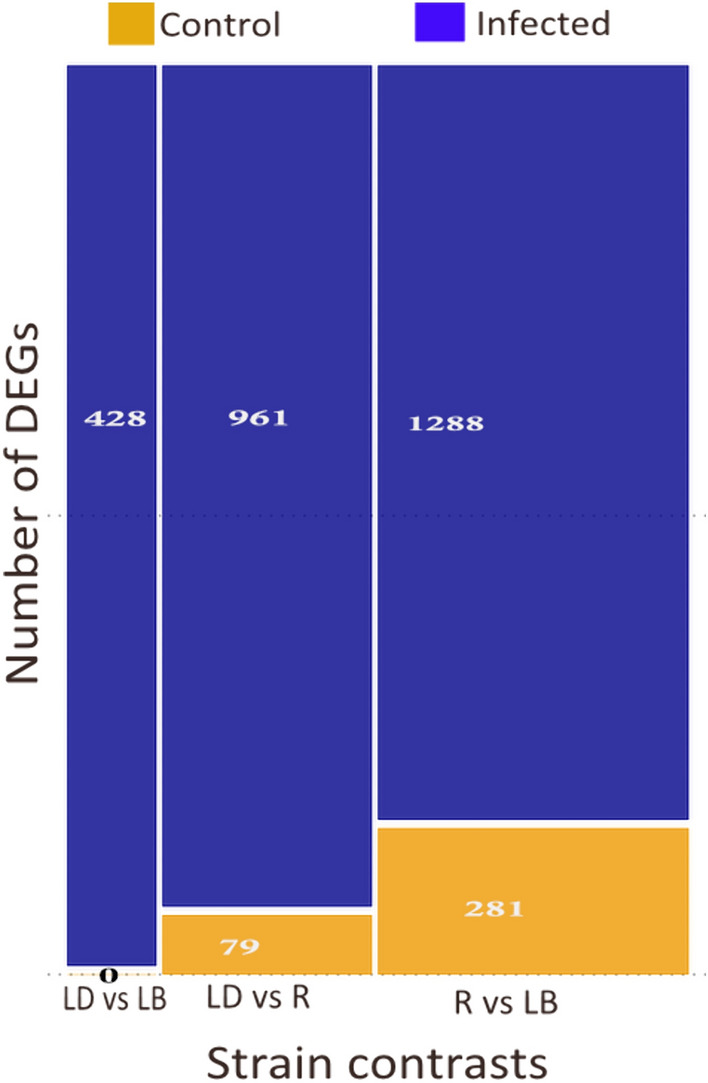
**Pairwise comparison of DEGs due to genotypic differences within the infection group.** Mosaic plot showing the number of differentially expressed genes (DEG) in the contrasts between the 3 chicken strains in infected (blue bar) and control (orange bar). There are no differences in the number of DEGs in the contrast between Lohmann Dual and Lohmann Brown in the control group.

Figure 6 shows the differences in the number of DEGs due to strain effects within infection status (i.e. strain effects in each infection group). The LD versus LB comparison revealed 428 DEGs in the infected birds, while no significant DEGs were quantified between the two strains in the uninfected control birds. Larger differences were observed between LD versus R birds, with 961 DEGs in the infected birds and 79 DEGs identified in the control group. Finally, the R and LB comparison revealed the two most divergent strains, with 281 DEGs identified in the control birds, while 1288 DEGs were found in the infected birds of the two strains.

#### Immune function-associated genes expressed in the liver

The list of DEGs found in both LD and R strains comprises several immune function-associated genes, including *IGLL1*, *CD74*, *IFNG*, and *NOS2* (Additional file 1). The list of genes that occur more frequently in the liver of these two strains also includes *FASLG* and *TNFSF8*, which belong to the tumour family of tumour necrosis factor. Additionally, the genes (*CCL5*, *CCL4*, *CCR5*, *CCL1*) that belong to the chemokine CC family and have inflammatory and chemokinetic properties are more abundant in the list of genes that are differentially expressed in the liver of infected chickens, as compared to the control group.

**Figure 7:**
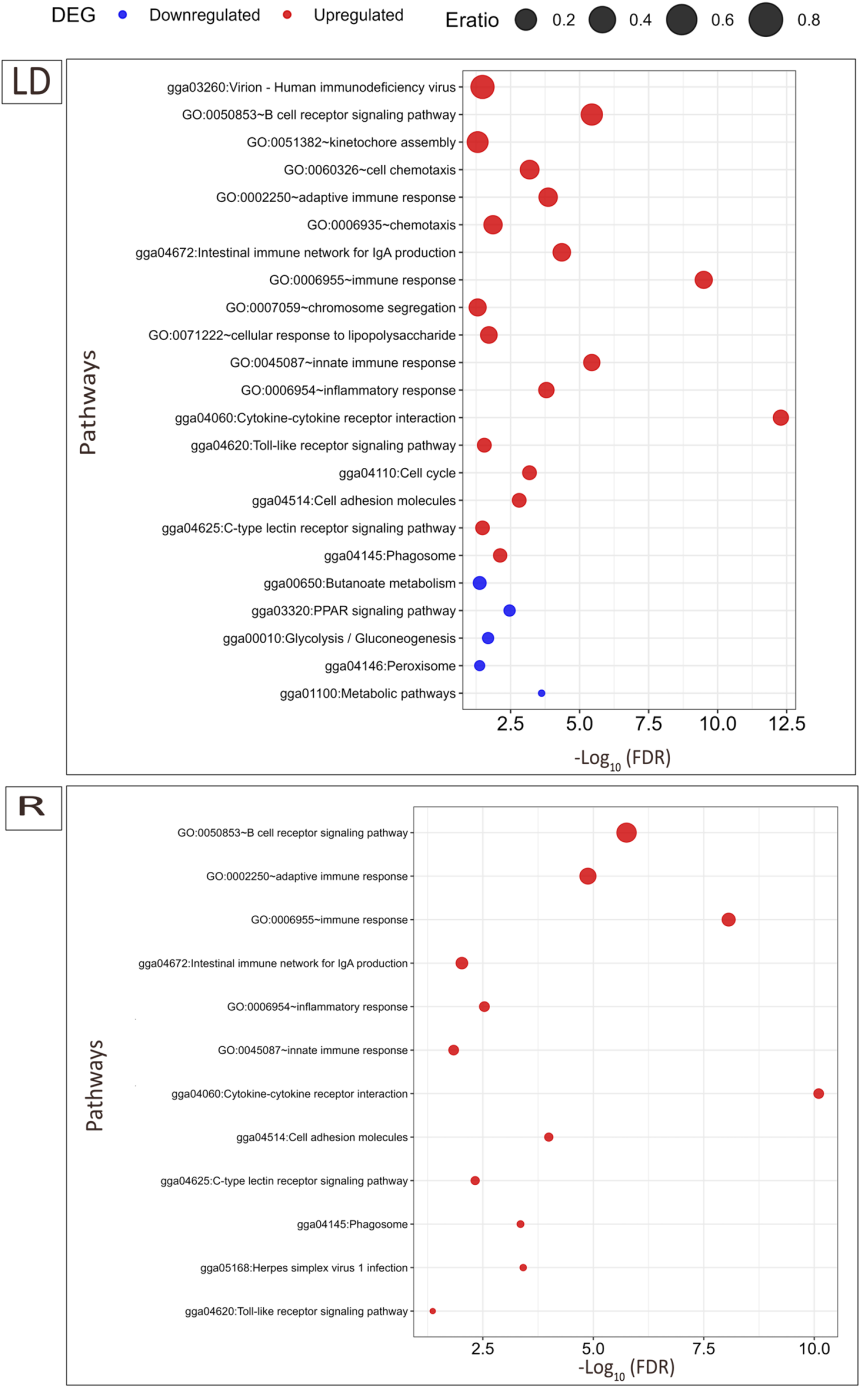
**Th genes in the hepatic of chickens infected with mixed parasites.** Differential expression levels of Th genes specific for immune functions in the liver of chickens infected with mixed parasites. Colour-filled bars: significant fold changes for each group of markers, FDR < 0.05. Positive/negative log_2_fold change value indicates up-regulated/downregulated expression in livers from infected birds compared with non-infected birds of **A** Lohmann brown strain, **B** Lohmann dual strain, **C** Ross strain. There are no significant DEGs assigned to Th in the Lohmann brown strain.

In examining the genes associated with Th1, Th2, Th17, and Treg immune responses in our dataset, we found 6 Th1 and 3 Th2 genes to have substantially higher expression in the infected birds of the LD strain. Similarly, 3 Th1 and 1 Th2 genes had considerably higher expression in the R strain than the respective control birds (Figure 7). In infected birds of the R strain, only one gene assigned to Th17 and Treg terms showed significant up-regulation, whereas, in the LD strain, four genes of each term were differentially expressed. None of the identified Th and Treg genes in the dataset were significantly expressed in the LB strain associated with the infection status (Figure 7).

#### Genes associated with metabolic processes expressed in the liver

Several lists of significantly differentially expressed genes (DEGs) in the LD strain included those associated with fatty acid and lipid metabolism that were down-regulated. Notable examples include *acetyl-CoA acetyltransferase 1* (*ACAT1*, log_2_FC = −0.88, FDR = 0.03), *fatty acid desaturase 2* (*FADS2*, log_2_FC = −0.91, FDR = 0.03), and *hydroxyacyl-CoA dehydrogenase* (*HADH*, log_2_FC = −0.98, FDR = 0.02). Both *ACAT1* and *HADH* are also involved in tryptophan metabolism and valine, leucine, and isoleucine degradation. Conversely, genes associated with the glycolytic process were up-regulated, including *aldolase C, fructose-bisphosphate* (*ALDOC*, log_2_FC = + 2.44, FDR < 0.01), *hexokinase 1* (*HK1*, log_2_FC = + 1.40, FDR < 0.01), *hexokinase 2* (*HK2*, log_2_FC = + 1.24, FDR = 0.03), and *phosphofructokinase, platelet* (*PFKP*, log_2_FC = + 1.91, FDR < 0.01).

### Gene set enrichment analysis

**Figure 8:**
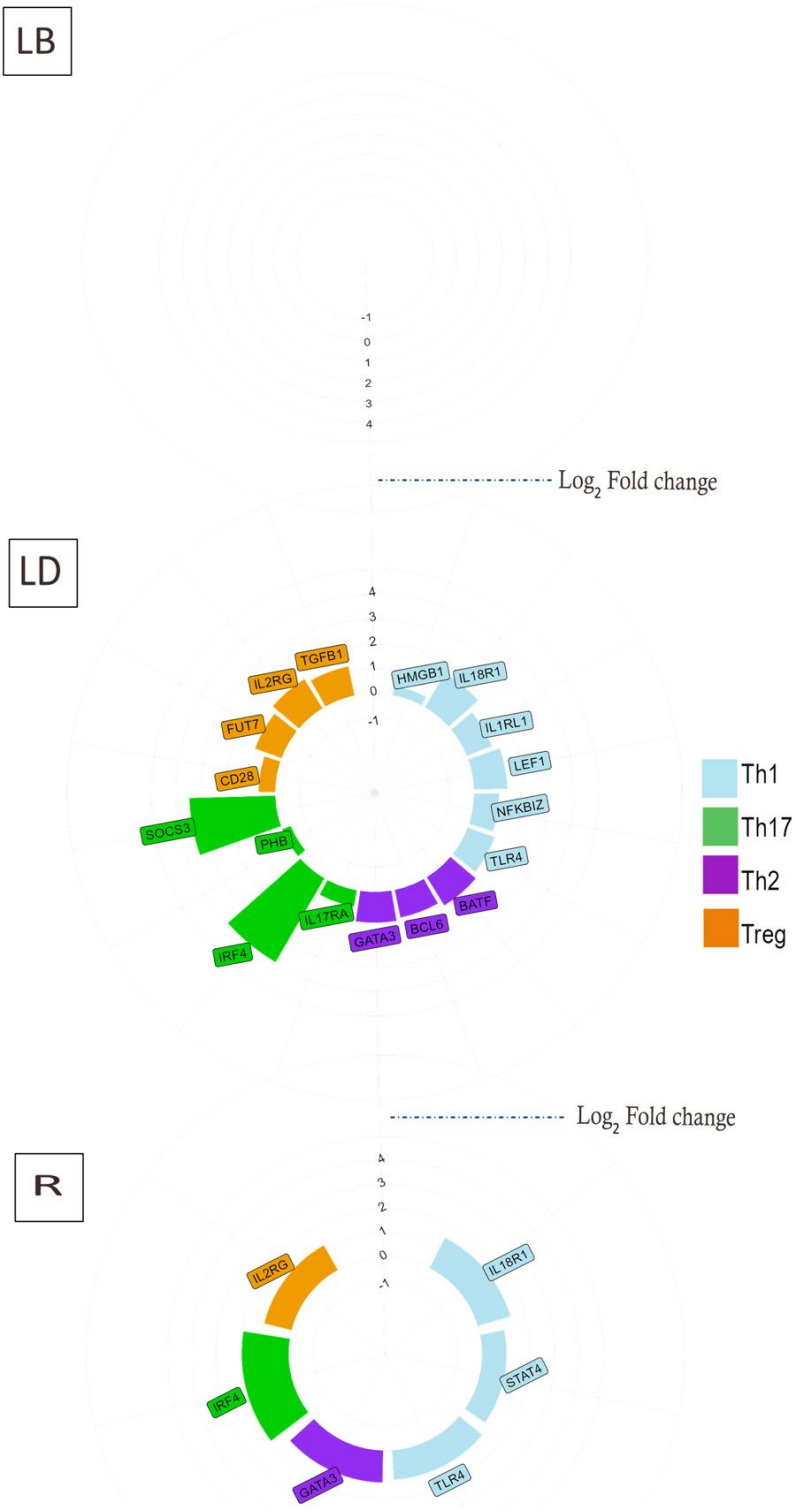
**Plot of significant GO and KEGG pathways enriched by significantly up-regulated genes (red dots) and downregulated genes (blue dots) due to infection effect in Lohmann dual (LD) and Ross (R) strain.** Eratio is the ratio of enrichment given by the number of observed genes divided by the number of expected genes from each GO or KEGG category.

Gene ontology (GO) term enrichment analysis revealed a functional understanding of significantly differentially expressed genes (log_2_FC > |1.0|, FDR < 0.05) in each of the strains as a result of the mixed infections. Figure 8 shows the results of GO term, biological process (BP), and KEGG pathway enrichment analysis for up-regulated DEGs associated with infection effects in LD and R birds. For the annotated up-regulated DEGs in LD birds, 7 BP-GO terms and 5 KEGG pathways were significantly enriched (FDR < 0.05). The terms *GO:00006955* ~ *immune response*, *GO:00006954* ~ *inflammatory response* and *gga04060: Cytokine-cytokine receptor interaction* were the most significantly enriched pathways in LD strains. Similar pathways were also the most enriched for the up-regulated DEGs in the R strain. The down-regulated DEGs were only enriched in the *gga01100: Metabolic pathways* and *gga00010: Glycolysis/Gluconeogenesis* pathways in LD strain (Figure 8). No GO term enrichment was observed among either up-regulated or down-regulated DEGs induced by the infections in LB animals.

## Discussion

The liver is a metabolically complex organ whose main functions may be compromised during exposure to environmental challenges, such as parasitic infections, particularly in strains selected for increased feed efficiency and fast growth performance. To cope with the challenges posed by infectious agents, especially when tissue damage occurs, the liver can initiate an appropriate inflammatory response that may result in metabolic remodelling [43]. We aimed to identify hepatic genes that signal immune and metabolic responses to mixed parasite infections in male birds of three different strains with distinct growth rates. The results demonstrate that the three strains differ considerably in their responses to mixed parasite infections, which may be attributed to the performance level of each strain.

Initially, the mixed infection had a negative impact on the birds’ performance, specifically their average feed intake. This impaired phenotypic trait could potentially explain the reduction in average daily weight gain. Reduced feed intake is a known response to nematode infections in chickens [44], particularly when histomonas infection is co-involved. The reduced feed intake observed in this study during infections could be linked to the activation of adaptive immune responses that increase cytokine levels. These cytokines are known to interact with neurochemicals involved in controlling feeding, which may lead to anorexia [5, 45].

It is well known that adaptive immune responses to ascarids in chickens begin within the first 2 weeks of infection [12, 21, 46] when the effects of ascarids on bird performance also start to become noticeable [11]. It is important to note that this study is part of a larger study where the birds were monitored for up to 9 wpi [13]. It has, therefore, already been established that a significant depression in growth becomes more pronounced from 3 wpi until the end of the experiment at 9 wpi, particularly in the R strain [13].

All the infected birds harboured worms at necropsy, confirming the presence of ascarid infections. Interestingly, the worm burdens with both *A. galli* and *H. gallinarum* were similar in all three strains. However, strain differences are known to occur in the worm burden at later stages, resulting from re-infections with the same nematodes [13]. Three of the infected birds (1 Ross and 2 LD strains) also showed liver tissue alterations that are characteristic of *H. meleagridis* infection [47], confirming the involvement of the protozoan parasites in the mixed infection as it occurs under natural conditions following *H. gallinarum* infection. This finding is corroborated by the increased concentration of the acute-phase protein AGP in the infected birds.

However, at the studied early stage of infection (2 WPI), the antibody titre against histomonas was not high enough to distinguish histomonosis in infected birds from non-infected ones, probably due to the non-instantaneous production of antibodies [48]. The histomonas counts in faeces samples did not differ among the three strains, implying a similar exposure to *H. meleagridis* infection in all three strains. This outcome is further supported by the similar concentrations of AGP in the three strains. However, a previous study found that livers of different chicken strains were differentially susceptible to the in vitro propagated clonal culture of *H. meleagridis* [49]. The alterations in the liver transcriptome of the infected birds cannot be solely attributed to nematode infections with *A. galli* and *H. gallinarum* or histomonas infections, but both to the mixed parasite infections and the performance level of each strain.

The initial findings of the differential gene expression analyses in this study revealed remarkable differences in the hepatic gene expression profiles of the three strains in response to mixed parasite infections. LB birds—male birds of a high-performing layer strain, thus representing a strain with low growth performance—showed minimal infection-related alterations at the transcriptional level. This outcome suggests a lower sensitivity to mixed parasite infections. The fact that female birds of the same strain (i.e. LB) are known to be more sensitive to mixed nematode infections than hens of the lower-performing LD strain [11] supports the hypothesis that high-performance pressure can lead to a prioritisation towards more vital functions of the organism [2].

In contrast to the male birds of the LB strain, the male birds of the intermediate (LD) and high-performing (R) strains exhibited a more pronounced molecular response in the liver caused by the mixed infection. Although basal hepatic gene expression differences were observed among non-infected birds, our findings show that infection significantly contributed to the majority of the differences in hepatic gene expression, with variations existing within the strains. Notably, the most substantial distinctions were observed between R (highest-performing strain) and the LB (lowest-performing strain), implying that the performance level of the host animal might be the most relevant driving force behind the differences. There were also differences between the high-performing R and the intermediate LD strains that were present; however, both strains had similar molecular responses, as indicated by the number of overlapping DEGs attributed to infection. This result implies a similarity in the response to infection between the two lines despite a marked difference in their performance. It is, therefore, plausible to propose that differences in response to mixed infection are more likely associated with a production level of the strain rather than inherent strain-specific factors. Although there were no differences in basal gene expression in the liver of either LD or LB strains, the gene expression profiles of both strains surprisingly and significantly differed in their response to mixed parasite infection. This difference emphasises the assumption that the response to infection feasibly depends on the production level of the strain, as male LD birds have a higher production pressure than male LB birds.

Nineteen genes were consistently differentially up-regulated in response to infections across all the strains, underscoring their potential significance, particularly in defence-related functions to mixed nematode infection. Among these was *IGLL1*, which plays a crucial role in producing immunoglobulin [50], ensuring the expression and secretion of antibodies, and contributing to antigen recognition. Notably, *IGLL1* emerges as a major target for poultry pathogens, including the Infectious Bursal Disease Virus (IBDV), and serves as one of the membrane binding sites during IBDV infection [50]. In this study, its increased expression in the liver of chickens exposed to ascarids and protozoan infection may suggest its substantial role in the pathophysiological responses to both infections.

*SOD3*, which belongs to part of the superoxide dismutase (SOD) protein family, is another gene of interest involved in responses to mixed parasite infection. *SOD3* acts as an antioxidant enzyme, converting superoxide radicals into hydrogen peroxide and oxygen [51]. This process serves to protect tissues from oxidative stress. The *SOD3*’s human ortholog is implicated in T cell infiltration, while its down-regulation during tumour development contributes to cancer progression [52]. Another gene, up-regulated in all three strains, *CHIA*, is involved in chitin degradation, a component found to be abundant in nematodes and the cell walls of fungi and arthropods [53]. The encoded protein facilitates chitin breakdown and stimulates the expression of interleukin 13 (IL-13), a key Th2 cytokine involved in local immune responses to helminth [54]. The increased expression of IL-13 in the jejunum and caeca during the early stage of mixed nematode infection (*A. galli* and *H. gallinarum*) has been associated with robust worm expulsion and the absence of parasite eggs [12, 21].

*RGL1*, located within the cytosol and involved in regulating catalytic activity, serves as a downstream effector protein in the Ras and Ral signalling pathways [55]. The Ras pathway is pivotal for transmitting signals in various cellular processes, encompassing cell proliferation, differentiation, and transformation [55]. *FABP3*, another gene up-regulated in the three strains, belongs to the fatty acid-binding proteins (FABP). FABPs, characterised by their small molecular weight and strong affinities for long-chain fatty acids, contribute to intracellular fatty acid movement, cell growth, differentiation, cellular signalling, gene transcription, and safeguarding enzymes from the detrimental effects of free fatty acids. Moreover, they may influence enzyme activity and signal transduction [56]. The *C7* gene encodes a serum glycoprotein, which collaborates with complement components *C5b*, *C6*, *C8*, and *C9* to form a membrane attack complex within the innate immune system’s terminal complement pathway. The protein houses a cholesterol-dependent cytolysin/membrane attack complex/perforin-like (CDC/MACPF) domain and belongs to a family of molecules engaged in host immunity. *C7* initiates the formation of the membrane attack complex by binding to the *C5b-C6* subcomplex, acting as a membrane anchor participating in immune defence mechanisms [57].

The infected LB birds exhibited significantly up-regulated *AvBD5*, *Avidin*, and *RBPJL* expression in the liver compared to the uninfected controls of the same strain. Interestingly, *AvBD5* is also significantly expressed in LD animals but not in the R strain. Beta-defensins, such as *AvBD5*, play a multifaceted role in immune regulation [58]. They are a family of antimicrobial peptides active against a wide range of microorganisms, including Gram-positive and Gram-negative bacteria, fungi, and yeast [59]. The activation of defensins against parasites is not well studied; however, they directly bind to chemokine receptors, inducing pro-inflammatory cytokine expression [60, 61]. Additionally, defensins demonstrate anti-inflammatory properties by mitigating LPS-induced inflammation [58].

In the LD strain, *AICDA* was significant, and in the Ross strain, *CRYBA2* and *SPI-C* were the most significantly expressed genes among the transcripts following infection. The *AICDA* gene encodes an RNA-editing deaminase crucial for three distinct immunoglobulin (Ig) diversification pathways: class switch recombination, somatic hypermutation, and Ig gene conversion [62]. *SPIC* is a novel ETS protein expressed in B lymphocytes. SPI-C was found to interact specifically with the C-terminus of STAT6, which led to the stimulation of IL4-induced IgE expression [63]. IL4 induction through activating the STAT 6 pathways has been shown to promote intestinal muscle contractility, enhancing worm expulsion [64, 65].

In the present study, particular emphasis was placed on the expression profiles of possible Th1, Th2, Th17 and Treg marker genes. This targeted analysis identified specific genes in the liver of infected chickens compared to the controls in both LD and R. Moreover, immune responses to ascarid infection were characterised by activation of the Th2 immune pathway. In contrast, histomonas infections were found to induce Th1 cytokines [28]. Typically, mono-infections with intracellular or extracellular pathogens result in a polarisation of the Th1 and Th2 pathways, but in our study with extracellular parasites, both pathways were activated simultaneously. This result is consistent with a study in pigs infected with *Ascaris suum*, the adult stages of which are found in the small intestine, while the larvae invade the liver during a migration phase. The mRNA expression of Th1 and Th2 markers was higher in the livers of infected pigs [20].

Similarly, both Th17 and Treg pathways have been identified as central in regulating adaptive immune responses during nematode infection [66]. The activation of the Th1 pathway may have been a response to co-infection with *H. meleagridis*, as observed in this study. Previous studies have indicated that *H. meleagridis* infections increase IFN-γ+CD4+ T cells in chickens [28].

Focusing on the most down-regulated genes in all the genotypes, the *FBF1* gene is the only down-regulated one in the LB strain. However, down-regulation of *PCK1*, *GABRA3*, and *NK6* genes is observed in the LD strain, whereas, within the R strain, *GHRHR*, *ATP2B2*, and *RALY* genes are the most down-regulated genes. *PCK1*, encoding phosphoenolpyruvate carboxylase (PEPCK), serves as a pivotal enzyme in gluconeogenesis, facilitating the rate-limiting step of phosphoenolpyruvate formation by decarboxylating oxaloacetate [67]. In birds, PEPCK-c is expressed in the liver, and its levels are highly responsive to factors such as nutrition, stress, and endocrine status [68]. The activities of PCK were significantly reduced following infection with reticuloendotheliosis-associated virus and reticuloendotheliosis virus [69].

Similarly, Dawson et al. [70], found *PCK1* to be down-regulated in the proximal colon of pigs infected with *Trichuris suis,* suggesting its implication in host response to infections. Conversely, the up-regulation of glycolytic genes, including *ALDOC*, *HK1*, and *PFKP*, may indicate a metabolic reprogramming, which may be due to the high energy demand necessitated by the response to infections, i.e. activation of immune responses. Although this depends on glucose availability, no differences in glucose plasma concentration were found in our study. This finding may be because the net effect of the regulation of genes in gluconeogenesis and glycolysis was zero.

The GO analysis indicated an enrichment of DEGs in several pathways. It revealed that the many up-regulated genes are involved in defence mechanisms, including inflammatory and stress responses. This finding suggests a more pronounced response to stress during infection in strains with certain production pressures. On the other hand, the down-regulation of genes primarily involved in gluconeogenesis may be attributable to decreased energy intake or a shift in the liver’s priorities towards immune response functions, potentially at the expense of metabolic processes. The up-regulation of immune-associated genes and the down-regulation of genes associated with metabolic processes in the liver appears to be a typical response to helminth infections, not just in chickens, as reported in this study, but in other species. A meta-analysis of transcriptome responses to helminth infections in different tissues of murine models shows that most up-regulated genes associated with the helminth infection are predominantly involved in inflammatory response antigen presentation.

In contrast, down-regulated genes are mainly involved in metabolic processes, including lipids and lipoprotein, cholesterol synthesis, glutathione, and vitamin D metabolism [71]. Similar outcomes were observed in sheep naturally infected with *Haemonchus contortus* [72] and pigs infected with *Ascaris suum* [73]. In line with the literature cited above, our results collectively corroborate that parasite infections lead to a shift in host liver functions between metabolism and defence. As our study focused on only one early time point (2 WPI), marking the onset of immune responses against infections, further investigation of later infection periods is recommended to understand the changes in physiological responses as the infection progresses. Additionally, examining other tissues (e.g., muscle and intestinal tissue) will enhance our understanding of the dynamics of metabolic and immune changes during helminth infection.

We identified distinct molecular responses to mixed parasite infections in chicken livers. During the early stage of infection, regardless of the chicken strains, defence regulation proved to be the most significant response to mixed parasite infections. Despite the large performance differences, the three strains responded with similar infection intensity but showed different hepatic molecular responses that were tuned to the performance levels. There was an indication of a switch in the hepatic function towards immune-related pathways, possibly at the expense of metabolic activities in response to ascarid and concurrent histomonas infections. When challenged with mixed parasite species, we conclude that the liver shifts its functions from metabolic to immune-related activities in chickens.

## Supplementary Information

**Additional file 1**. **Significantly expressed genes in strains of chickens exposed to mixed parasite infections.** A: List of genes significantly expressed in infected chickens of all three strains; B: List of genes significantly expressed in infected chickens of LD and R strains; Gene_ID: ensemble gene identifier; FDR: false discovery rate adjusted *P*-value. Log_2_FC: fold change.

## Data Availability

The datasets supporting the conclusions of this article are available in the Zenodo repository [10.5281/zenodo.13169113].

## Abbreviations

ADG: average daily weight gain
AFI: average daily feed intake
DAVID: database for annotation, visualization and integrated discovery
DEG: differentially expressed genes
FC: fold change
FDR: false discovery rate
GO: Gene Ontology
KEGG: Kyoto Encyclopedia of Genes and Genome
LB: Lohmann Brown
LD: Lohmann Dual
R: Ross
wpi: week post-infection
