# Supplementary material for: Hepatic transcriptomic analysis reveals differential regulation of metabolic and immune pathways in three strains of chickens with distinct growth rates exposed to mixed parasite infections
Source: Vet Res. 2024 Sep 28;55:125. doi: 10.1186/s13567-024-01378-8 (PMC11439216; doi:10.1186/s13567-024-01378-8)
Supplement: Supplementary file 1 — Additional file 1. Significantly expressed genes in strains of chickens exposed to mixed parasite infections. A: List of genes significantly expressed in infected chickens of all three strains; B: List of genes significantly expressed in infected chickens of LD and R strains; Gene_ID: ensemble gene identifier; FDR: false discovery rate adjusted P-value. Log2FC: fold change. [file 13567_2024_1378_MOESM1_ESM.docx]

A : List of genes significantly expressed in infected chickens of all

three strains

| All strains | | | |
| --- | --- | --- | --- |
| Gene | Gene_name | FDR | Log_2_FC |
| ENSGALG00000004728 | RGL1 | 6.67E-04 | 0.718584 |
| ENSGALG00000005937 |  | 4.10E-10 | 7.561804 |
| ENSGALG00000008940 |  | 0.001855214 | 5.047924 |
| ENSGALG00000014835 | C7 | 0.001481719 | 0.980008 |
| ENSGALG00000018557 | SOD3 | 0.001200873 | 2.007116 |
| ENSGALG00000021139 | IGLL1 | 8.61E-11 | 7.376925 |
| ENSGALG00000023760 | CHIA | 0.020998791 | 1.707992 |
| ENSGALG00000027789 | RAB37 | 0.00111129 | 1.473713 |
| ENSGALG00000030075 |  | 8.04E-05 | 2.264125 |
| ENSGALG00000030653 |  | 3.10E-07 | 7.021563 |
| ENSGALG00000031887 |  | 0.001791587 | 1.925737 |
| ENSGALG00000031998 |  | 2.41E-04 | 2.351949 |
| ENSGALG00000032344 |  | 4.00E-05 | 2.096908 |
| ENSGALG00000034721 |  | 8.29E-10 | 8.450146 |
| ENSGALG00000037050 | FABP3 | 1.06E-04 | 4.187443 |
| ENSGALG00000039268 |  | 5.21E-07 | 6.929573 |
| ENSGALG00000042285 |  | 8.61E-11 | 8.37656 |
| ENSGALG00000043610 |  | 6.73E-04 | 1.883097 |
| ENSGALG00000043742 |  | 1.97E-10 | 8.217464 |

B : List of genes significantly expressed in infected chickens of LD and R strains

| LD vs R strain | | | |
| --- | --- | --- | --- |
| Gene ID | Gene_name | FDR | Log_2_FC |
| ENSGALG00000000141 | BLB1 | 0.002754082 | 1.329249454 |
| ENSGALG00000000158 | MHCDMA | 0.001544502 | 1.910458136 |
| ENSGALG00000000352 | TIMM44 | 0.026652147 | -0.233453853 |
| ENSGALG00000000466 | LCK | 5.61E-04 | 1.509528197 |
| ENSGALG00000000504 | ETV7 | 0.026478885 | 1.551409057 |
| ENSGALG00000000529 | LIMD2 | 0.001133228 | 1.434134023 |
| ENSGALG00000000544 | RAB44 | 2.67E-04 | 1.84904721 |
| ENSGALG00000000589 | BTN1A1 | 5.26E-04 | 1.10027713 |
| ENSGALG00000000619 | ANGPTL4 | 0.041211221 | -1.570880162 |
| ENSGALG00000000681 | PAK1 | 0.002410442 | 1.22293055 |
| ENSGALG00000000731 | THEMIS2 | 0.005677203 | 1.112054971 |
| ENSGALG00000000775 | CTSS | 0.001273296 | 0.947659385 |
| ENSGALG00000000783 | PTAFR | 0.003274344 | 1.633212072 |
| ENSGALG00000001094 | ADGRD2 | 4.62E-04 | 2.915209102 |
| ENSGALG00000001149 | ADGRG5 | 0.014396382 | 1.390602212 |
| ENSGALG00000001161 | FLI1 | 0.001600301 | 0.781659901 |
| ENSGALG00000001256 | NLRC5 | 0.033375298 | 0.649368612 |
| ENSGALG00000001304 |  | 0.001678007 | 2.022688552 |
| ENSGALG00000001373 | TRAF3IP3 | 0.001273296 | 0.953716401 |
| ENSGALG00000001412 | SEMA7A | 0.009552071 | 1.026233752 |
| ENSGALG00000001486 | ZAP70 | 1.71E-05 | 2.060681548 |
| ENSGALG00000001564 | ATP2A3 | 3.58E-04 | 1.466338636 |
| ENSGALG00000001569 | RHOC | 0.001288148 | -0.608409207 |
| ENSGALG00000001571 | MYO1F | 0.002809778 | 1.495006342 |
| ENSGALG00000001587 | MRPL40 | 0.028783548 | -0.305530092 |
| ENSGALG00000001603 | NARF | 0.001791587 | 1.382338598 |
| ENSGALG00000001605 | PPM1J | 0.026329575 | 0.733441262 |
| ENSGALG00000001607 | CYBC1 | 2.91E-04 | 1.480555763 |
| ENSGALG00000001797 | GPSM1 | 0.011314803 | 0.907108386 |
| ENSGALG00000001882 | TNFRSF18 | 2.93E-04 | 1.803539543 |
| ENSGALG00000001894 |  | 2.51E-04 | 2.053397775 |
| ENSGALG00000002102 |  | 0.0069491 | 1.955914539 |
| ENSGALG00000002112 | CSF3R | 0.017261404 | 2.341398083 |
| ENSGALG00000002113 | LCP2 | 3.32E-04 | 1.602723092 |
| ENSGALG00000002160 | B2M | 2.77E-04 | 1.403991826 |
| ENSGALG00000002167 | PATL2 | 0.001960629 | 1.62221456 |
| ENSGALG00000002192 | PTPRC | 2.00E-04 | 2.010150908 |
| ENSGALG00000002207 | ATP6V1G3 | 0.003251106 | 2.541846374 |
| ENSGALG00000002263 | KLHL6 | 2.36E-04 | 1.251800564 |
| ENSGALG00000002329 | CCL1 | 1.35E-04 | 2.616365152 |
| ENSGALG00000002335 | DOCK3 | 0.015629262 | 1.998212848 |
| ENSGALG00000002525 | RASGEF1A | 2.11E-04 | 2.419527175 |
| ENSGALG00000002570 |  | 6.73E-04 | 2.693575233 |
| ENSGALG00000002583 | PIK3CD | 0.001094781 | 1.524378696 |
| ENSGALG00000002615 | DEF6 | 4.06E-04 | 1.230873034 |
| ENSGALG00000002728 | SLC16A3 | 0.007753333 | 3.4120314 |
| ENSGALG00000002786 | PSTPIP1 | 5.35E-04 | 1.834119751 |
| ENSGALG00000002816 | STK10 | 0.005805865 | 0.720007306 |
| ENSGALG00000002982 | SLC2A6 | 0.011379789 | 1.336619532 |
| ENSGALG00000003076 | FASLG | 0.011623806 | 1.200730992 |
| ENSGALG00000003106 |  | 0.005746099 | 1.509777436 |
| ENSGALG00000003113 | RGS14 | 0.002237425 | 1.584468877 |
| ENSGALG00000003136 | IKZF2 | 3.84E-04 | 1.716578769 |
| ENSGALG00000003160 | CERS1 | 0.007394767 | 0.806868008 |
| ENSGALG00000003161 | GDF3 | 0.008679969 | 0.784064531 |
| ENSGALG00000003185 | SCPEP1 | 0.009042333 | 0.726882017 |
| ENSGALG00000003196 | RNASE6 | 2.41E-04 | 2.342334547 |
| ENSGALG00000003213 |  | 0.029255455 | 1.154085218 |
| ENSGALG00000003217 | LITAF | 4.42E-04 | 2.529757594 |
| ENSGALG00000003349 | PLEKHA2 | 0.00112454 | 1.515514315 |
| ENSGALG00000003361 | TREM-B1 | 0.012839849 | 1.008027459 |
| ENSGALG00000003499 | MAST3 | 0.007075613 | 1.267857668 |
| ENSGALG00000003651 | NT5C3B | 0.002121527 | -0.581577298 |
| ENSGALG00000003670 | MAFB | 2.51E-04 | 1.320328635 |
| ENSGALG00000003750 | PLCG1 | 2.99E-04 | 0.917358863 |
| ENSGALG00000003789 | FAM78A | 0.003573061 | 1.330264758 |
| ENSGALG00000003792 | FYB | 6.79E-04 | 1.443790172 |
| ENSGALG00000003802 | OTUD7A | 0.003133651 | 0.953484063 |
| ENSGALG00000003837 | ADCY7 | 0.003898849 | 1.169398694 |
| ENSGALG00000003841 |  | 0.003397645 | 1.920031155 |
| ENSGALG00000003842 | GHRH | 1.73E-04 | 2.429127442 |
| ENSGALG00000003857 | ITK | 8.04E-05 | 1.722785181 |
| ENSGALG00000003863 | P2RX7 | 0.001882012 | 1.171895963 |
| ENSGALG00000003919 | MRPL22 | 0.035489665 | -0.257955919 |
| ENSGALG00000004088 | FNBP1 | 0.0019832 | 0.659427525 |
| ENSGALG00000004111 | GPR174 | 1.24E-04 | 2.040916422 |
| ENSGALG00000004113 |  | 0.023675637 | 1.329515497 |
| ENSGALG00000004167 | SRGN | 6.96E-04 | 2.152839103 |
| ENSGALG00000004222 | HK1 | 9.23E-04 | 1.383176772 |
| ENSGALG00000004252 |  | 0.025296476 | 0.856154217 |
| ENSGALG00000004257 | TSPAN15 | 0.002581213 | 2.452042419 |
| ENSGALG00000004266 | TNFAIP8L1 | 0.005337995 | 0.839253561 |
| ENSGALG00000004276 | CHST12 | 0.012732451 | 0.679651965 |
| ENSGALG00000004336 | INPPL1 | 0.004903133 | 1.609006257 |
| ENSGALG00000004398 | CARD11 | 0.001273296 | 1.275200764 |
| ENSGALG00000004400 | SPECC1 | 4.62E-04 | 1.732251618 |
| ENSGALG00000004425 | SCAMP1 | 0.002910478 | 0.519520455 |
| ENSGALG00000004519 | TRPV2 | 0.0040152 | 0.707130258 |
| ENSGALG00000004594 | CD74 | 0.002783665 | 1.29710906 |
| ENSGALG00000004613 | P2RX5 | 8.24E-04 | 1.500871364 |
| ENSGALG00000004662 | DISP3 | 0.021902641 | 0.507177117 |
| ENSGALG00000004700 | NCF2 | 0.004979032 | 2.752853117 |
| ENSGALG00000004771 | C1QB | 4.62E-04 | 1.068958921 |
| ENSGALG00000004901 | CORO1C | 8.76E-04 | 1.275363942 |
| ENSGALG00000004916 | DRC3 | 4.73E-04 | 2.166590965 |
| ENSGALG00000004958 | BTK | 0.004210727 | 1.22261524 |
| ENSGALG00000004980 | KCNMA1 | 9.89E-04 | 1.305631497 |
| ENSGALG00000005030 | DOCK10 | 2.03E-04 | 1.157133112 |
| ENSGALG00000005052 |  | 0.026414922 | 1.818402559 |
| ENSGALG00000005062 | FOXN4 | 0.031911451 | 0.842130541 |
| ENSGALG00000005065 | PLA2G4A | 0.009429623 | 1.430004801 |
| ENSGALG00000005086 | PLAU | 0.035836848 | 0.716346912 |
| ENSGALG00000005094 | GRAP | 2.19E-04 | 1.889942197 |
| ENSGALG00000005257 |  | 2.93E-04 | 1.395085528 |
| ENSGALG00000005367 | MRPS22 | 0.048763708 | -0.307351739 |
| ENSGALG00000005505 | PIK3CB | 0.008937639 | 0.95591903 |
| ENSGALG00000005543 |  | 0.003233902 | 2.222940567 |
| ENSGALG00000005544 | GPR55 | 3.95E-04 | 4.358448508 |
| ENSGALG00000005547 | PIK3AP1 | 0.003898849 | 0.695155287 |
| ENSGALG00000005589 | SYNGR3 | 5.91E-04 | 1.503358436 |
| ENSGALG00000005601 |  | 0.009338535 | 1.380846965 |
| ENSGALG00000005638 | IL2RG | 5.65E-04 | 1.377740683 |
| ENSGALG00000005644 | MYO1G | 4.45E-04 | 1.796294536 |
| ENSGALG00000005685 | KSR1 | 0.00336772 | 0.796630144 |
| ENSGALG00000005725 | CSF1R | 0.002115952 | 0.914409701 |
| ENSGALG00000005809 | Sep-02 | 4.43E-05 | 1.734776174 |
| ENSGALG00000005823 | HDLBP | 0.043847513 | -0.338798465 |
| ENSGALG00000005964 |  | 0.006654619 | 1.328621117 |
| ENSGALG00000005983 | SMARCB1 | 0.048327839 | -0.162613431 |
| ENSGALG00000006017 | DOCK11 | 0.001746994 | 1.112595683 |
| ENSGALG00000006152 |  | 0.014610657 | 1.013261304 |
| ENSGALG00000006165 | WDFY4 | 1.70E-04 | 1.729457077 |
| ENSGALG00000006229 |  | 0.001711182 | 1.618601646 |
| ENSGALG00000006237 | PKN2 | 0.001834026 | 0.529588126 |
| ENSGALG00000006318 | IL21R | 4.35E-05 | 3.264376894 |
| ENSGALG00000006334 | LAP3 | 0.024506265 | -0.501952706 |
| ENSGALG00000006352 | CH25H | 0.00399956 | 2.346469369 |
| ENSGALG00000006378 | LIPA | 0.0019832 | 0.865982047 |
| ENSGALG00000006388 | IL16 | 3.57E-05 | 2.030841661 |
| ENSGALG00000006402 |  | 0.00166308 | 1.247338766 |
| ENSGALG00000006527 | CRTAM | 4.11E-05 | 2.041290155 |
| ENSGALG00000006583 | LSP1 | 8.06E-05 | 2.063903883 |
| ENSGALG00000006779 | PDZD3 | 0.037987266 | 0.718721118 |
| ENSGALG00000006785 | IRF1 | 0.001913365 | 2.007247455 |
| ENSGALG00000006939 | PFKP | 4.22E-04 | 1.913866916 |
| ENSGALG00000006992 | MMP9 | 0.044186016 | 2.320668573 |
| ENSGALG00000007001 | TLR4 | 0.02596795 | 1.159939221 |
| ENSGALG00000007028 |  | 5.20E-04 | 1.238009445 |
| ENSGALG00000007059 | IGSF6 | 4.32E-04 | 1.017792696 |
| ENSGALG00000007078 | ENTPD1 | 0.006288296 | 0.875325235 |
| ENSGALG00000007093 | CYTH1 | 0.004169429 | 0.825311905 |
| ENSGALG00000007121 | TNFSF8 | 0.001426919 | 2.710774851 |
| ENSGALG00000007123 |  | 0.007895467 | 1.730830878 |
| ENSGALG00000007141 |  | 0.001426919 | 1.245372863 |
| ENSGALG00000007171 |  | 0.001402311 | 1.514914628 |
| ENSGALG00000007186 | TMEM268 | 0.004807896 | 1.028568039 |
| ENSGALG00000007210 |  | 0.004504466 | 1.917137536 |
| ENSGALG00000007216 | TVP23A | 0.014920647 | 1.396771433 |
| ENSGALG00000007248 |  | 0.008047806 | 1.280055808 |
| ENSGALG00000007416 | CD3E | 4.35E-05 | 1.760100598 |
| ENSGALG00000007418 | CD3D | 2.37E-04 | 1.639237837 |
| ENSGALG00000007511 | ITGB2 | 7.03E-04 | 1.600656656 |
| ENSGALG00000007522 |  | 8.15E-04 | 1.000928561 |
| ENSGALG00000007546 |  | 3.73E-06 | 2.053940882 |
| ENSGALG00000007609 | ARHGAP19 | 0.001135413 | 2.4232371 |
| ENSGALG00000007651 | STAT1 | 0.014289259 | 1.227672874 |
| ENSGALG00000007744 | CASS4 | 4.62E-04 | 1.468571284 |
| ENSGALG00000007841 | DNAH7 | 0.047746497 | 0.690451109 |
| ENSGALG00000007845 | REL | 0.001951923 | 1.038849889 |
| ENSGALG00000007877 | PIP4K2A | 7.14E-04 | 1.156157762 |
| ENSGALG00000007920 | PRR5L | 0.024037195 | 1.245062287 |
| ENSGALG00000007924 | STK17B | 1.24E-04 | 1.428670092 |
| ENSGALG00000007963 | NFATC2 | 0.002810728 | 1.101116597 |
| ENSGALG00000007996 | ANKRD44 | 0.001000971 | 0.906988925 |
| ENSGALG00000007999 | MRPS7 | 0.047764133 | -0.261001192 |
| ENSGALG00000008088 | ACSL4 | 2.26E-04 | 1.083327649 |
| ENSGALG00000008127 | SPI1 | 0.001348977 | 1.502450707 |
| ENSGALG00000008166 | TLR15 | 0.007897643 | 3.115860462 |
| ENSGALG00000008257 | MERTK | 9.47E-04 | 1.042801982 |
| ENSGALG00000008267 | IL5RA | 0.001305445 | 3.510865115 |
| ENSGALG00000008280 | PMPCB | 0.016641873 | -0.28924893 |
| ENSGALG00000008306 | FGL2 | 4.08E-04 | 1.65736435 |
| ENSGALG00000008340 | FES | 0.007928489 | 1.056655294 |
| ENSGALG00000008447 | SH2D1A | 5.65E-04 | 1.836844959 |
| ENSGALG00000008623 | TOR4A | 0.005699462 | 0.754590568 |
| ENSGALG00000008656 | ICOS | 0.012469473 | 1.139478908 |
| ENSGALG00000008660 | CST7 | 0.032890215 | 1.090446803 |
| ENSGALG00000008666 | CTLA4 | 9.86E-06 | 5.193870951 |
| ENSGALG00000008735 | BFSP1 | 0.037823288 | 0.857391998 |
| ENSGALG00000008756 | PLEK | 0.007946311 | 1.299182019 |
| ENSGALG00000008757 | CNRIP1 | 6.53E-04 | 1.739613786 |
| ENSGALG00000008759 | LAMP3 | 3.47E-04 | 2.128270453 |
| ENSGALG00000008881 | RGS3 | 0.022816127 | 0.534324241 |
| ENSGALG00000008911 | RNF208 | 0.009143909 | 1.915054742 |
| ENSGALG00000008950 | EHD4 | 0.004472906 | 1.205075093 |
| ENSGALG00000008999 | AFAP1L2 | 0.00744286 | 0.759252263 |
| ENSGALG00000009052 | LBH | 0.047029387 | 0.781531344 |
| ENSGALG00000009369 | PRDX3 | 0.048656842 | -0.460866542 |
| ENSGALG00000009422 | RGS10 | 0.005434358 | 1.515583889 |
| ENSGALG00000009450 | DUSP10 | 3.09E-04 | 1.496049179 |
| ENSGALG00000009479 |  | 0.016713808 | 1.597008594 |
| ENSGALG00000009520 | Mar-01 | 0.001133913 | 1.933550223 |
| ENSGALG00000009621 | ACTB | 0.007748654 | 1.133552554 |
| ENSGALG00000009740 | RASGRP1 | 8.99E-04 | 1.336789309 |
| ENSGALG00000009823 | PTPRE | 0.003253388 | 1.248186347 |
| ENSGALG00000009855 | TBC1D9 | 0.030833271 | -0.828634004 |
| ENSGALG00000009864 | TRAF5 | 3.83E-06 | 2.201427728 |
| ENSGALG00000009945 | CPM | 0.00292601 | 1.285625217 |
| ENSGALG00000010068 | KCNK17 | 0.002973736 | 1.323015264 |
| ENSGALG00000010119 | CDKL2 | 0.031325928 | 0.863293359 |
| ENSGALG00000010156 |  | 8.04E-05 | 1.539699398 |
| ENSGALG00000010166 | VLDLR | 3.26E-04 | 1.591661831 |
| ENSGALG00000010224 | GLIPR1L | 0.004630279 | 1.030695486 |
| ENSGALG00000010237 | NPC2 | 0.00856623 | 0.880008908 |
| ENSGALG00000010311 | NAV3 | 3.50E-04 | 1.560116305 |
| ENSGALG00000010331 | MME | 3.88E-04 | 3.907980536 |
| ENSGALG00000010337 | GPR149 | 2.33E-04 | 3.236886955 |
| ENSGALG00000010582 | PPA2 | 0.005900658 | -0.450305794 |
| ENSGALG00000010595 | GPR65 | 4.33E-05 | 1.5054558 |
| ENSGALG00000010636 | PRIMPOL | 0.005805865 | 0.666172241 |
| ENSGALG00000010702 | ADSS | 0.002890414 | 1.362222674 |
| ENSGALG00000010703 | DGLUCY | 0.035894622 | -0.997019426 |
| ENSGALG00000010748 | EXO1 | 7.45E-04 | 3.48511469 |
| ENSGALG00000010770 | SCIN | 4.46E-04 | 1.778839056 |
| ENSGALG00000010806 | RIN3 | 0.001131867 | 1.291490331 |
| ENSGALG00000010881 | ASB2 | 0.004244545 | 1.394780966 |
| ENSGALG00000010960 | GALNT3 | 0.002201387 | 0.979420331 |
| ENSGALG00000011116 | VRK1 | 4.29E-04 | 0.98570392 |
| ENSGALG00000011203 | HPSE | 0.00180017 | 1.838423051 |
| ENSGALG00000011209 | EVL | 4.66E-04 | 1.637926369 |
| ENSGALG00000011222 | IL12RB2 | 3.63E-04 | 1.237835365 |
| ENSGALG00000011248 | PLCL2 | 1.88E-04 | 1.722311689 |
| ENSGALG00000011309 | PLXNC1 | 0.009513241 | 0.777660406 |
| ENSGALG00000011404 | HOPX | 2.77E-04 | 1.756317423 |
| ENSGALG00000011446 | TNFAIP2 | 0.003925246 | 1.095213091 |
| ENSGALG00000011488 | CMTM7 | 5.77E-04 | 1.492730357 |
| ENSGALG00000011489 | T | 0.007082731 | 2.031339293 |
| ENSGALG00000011551 | JCHAIN | 6.67E-06 | 4.128042258 |
| ENSGALG00000011608 | INF2 | 0.007263786 | 0.778646178 |
| ENSGALG00000011614 | ANO4 | 0.008621012 | 1.59426424 |
| ENSGALG00000011646 | PLD4 | 0.023142001 | 0.999356396 |
| ENSGALG00000011684 | STAP1 | 0.005724978 | 1.667212194 |
| ENSGALG00000011733 | CCR2 | 0.001882012 | 1.890667001 |
| ENSGALG00000011735 | XCR1 | 0.002791156 | 1.427778715 |
| ENSGALG00000011738 | ARHGDIB | 3.09E-04 | 1.459636698 |
| ENSGALG00000011803 | EMP1 | 0.009014265 | 2.498379872 |
| ENSGALG00000011872 |  | 0.003397645 | 1.662236147 |
| ENSGALG00000011889 |  | 3.14E-05 | 1.624240903 |
| ENSGALG00000011961 | TNIP3 | 8.04E-05 | 3.818184234 |
| ENSGALG00000012056 |  | 0.002642716 | 1.243584541 |
| ENSGALG00000012076 | TIFA | 3.88E-04 | 1.807029052 |
| ENSGALG00000012119 | MARCO | 0.019902286 | 0.819930535 |
| ENSGALG00000012165 | DLGAP5 | 1.81E-04 | 2.908772625 |
| ENSGALG00000012173 | LGALS3 | 0.001808856 | 1.467011127 |
| ENSGALG00000012292 | BANK1 | 0.001558814 | 1.725482559 |
| ENSGALG00000012317 | ADGB | 0.010173355 | 1.661514796 |
| ENSGALG00000012421 | ARHGAP15 | 1.24E-04 | 1.585648543 |
| ENSGALG00000012422 |  | 2.91E-04 | 1.89634536 |
| ENSGALG00000012454 | CYTH4 | 0.008047806 | 0.994564045 |
| ENSGALG00000012456 | RAC2 | 0.001841975 | 1.313214975 |
| ENSGALG00000012462 | KIF5C | 0.00420664 | 1.432719687 |
| ENSGALG00000012472 | IL2RB | 4.25E-04 | 1.679285421 |
| ENSGALG00000012543 | GPD2 | 0.008190271 | 1.004694223 |
| ENSGALG00000012545 | CYTIP | 3.48E-04 | 1.775103924 |
| ENSGALG00000012595 | AGTPBP1 | 3.30E-04 | 1.252170157 |
| ENSGALG00000012618 | FANCC | 9.77E-04 | 2.14234266 |
| ENSGALG00000012801 | LY86 | 0.038674331 | 0.463608754 |
| ENSGALG00000012823 | TRIM24 | 0.037987266 | 0.481099848 |
| ENSGALG00000012830 | IRF4 | 4.92E-07 | 3.900473763 |
| ENSGALG00000012932 | FGD4 | 0.003254773 | 1.177703786 |
| ENSGALG00000012965 | FAM105A | 2.26E-04 | 1.487155607 |
| ENSGALG00000012969 |  | 0.024986245 | 1.560370173 |
| ENSGALG00000013086 | IKZF1 | 5.35E-04 | 1.756548176 |
| ENSGALG00000013189 | LPCAT1 | 0.010397158 | 0.647128367 |
| ENSGALG00000013218 | C3AR1 | 0.007938217 | 1.100795907 |
| ENSGALG00000013253 | INSRR | 0.003777264 | 0.960037189 |
| ENSGALG00000013409 |  | 0.018244822 | 1.765470941 |
| ENSGALG00000013481 | IKZF3 | 2.57E-04 | 1.945889079 |
| ENSGALG00000013546 |  | 2.75E-07 | 2.651497775 |
| ENSGALG00000013548 | GZMA | 3.10E-07 | 4.889090242 |
| ENSGALG00000013625 |  | 0.025252421 | 1.105208139 |
| ENSGALG00000013741 | SYTL3 | 3.38E-04 | 1.358122542 |
| ENSGALG00000013747 | TAGAP | 0.001940523 | 1.782979437 |
| ENSGALG00000014054 | GRK6 | 0.016966123 | 0.509952204 |
| ENSGALG00000014124 | TEC | 0.01400655 | 0.901204845 |
| ENSGALG00000014127 | TXK | 0.010568855 | 1.019935981 |
| ENSGALG00000014128 | A4GALT | 0.042811847 | 1.116626873 |
| ENSGALG00000014203 | PARVG | 0.001558814 | 1.365725072 |
| ENSGALG00000014362 | CD86 | 0.021066362 | 1.412885428 |
| ENSGALG00000014412 | CSTA | 0.001465705 | 2.6670083 |
| ENSGALG00000014453 | TENT5C | 9.85E-07 | 3.911981835 |
| ENSGALG00000014455 | LPAR5 | 0.004081547 | 1.556332648 |
| ENSGALG00000014477 | CD4 | 0.001529538 | 1.285491907 |
| ENSGALG00000014508 | CD38 | 3.81E-04 | 0.99828087 |
| ENSGALG00000014561 | PTPN6 | 0.009205979 | 0.646610591 |
| ENSGALG00000014585 |  | 2.40E-04 | 2.458368753 |
| ENSGALG00000014668 | MCTP1 | 0.001265843 | 0.9086943 |
| ENSGALG00000014688 | ZYX | 0.003831882 | 0.750201837 |
| ENSGALG00000014727 | PDE4D | 0.003831882 | 1.724175459 |
| ENSGALG00000014750 |  | 0.001586808 | 1.623546517 |
| ENSGALG00000014754 |  | 2.76E-06 | 2.546224479 |
| ENSGALG00000014801 | NDC80 | 1.35E-04 | 2.583861677 |
| ENSGALG00000014821 | THEMIS | 0.001375427 | 1.684990753 |
| ENSGALG00000014846 | OXCT1 | 8.42E-04 | 1.168630696 |
| ENSGALG00000014880 |  | 0.005805865 | 1.063482173 |
| ENSGALG00000014933 | HEXB | 0.00743782 | 0.527388396 |
| ENSGALG00000015022 | FYN | 0.007151571 | 1.108489561 |
| ENSGALG00000015032 |  | 2.57E-04 | 2.788995543 |
| ENSGALG00000015126 | TRPM3 | 0.004985216 | 3.725339265 |
| ENSGALG00000015132 | CDH2 | 0.002605007 | 2.410557617 |
| ENSGALG00000015192 |  | 0.034217533 | 2.013083477 |
| ENSGALG00000015216 | SYK | 1.21E-04 | 1.754545052 |
| ENSGALG00000015235 | XCL1 | 1.21E-05 | 2.153132273 |
| ENSGALG00000015348 | ALCAM | 8.15E-04 | 1.397919753 |
| ENSGALG00000015362 | TRAT1 | 0.016663616 | 1.207503291 |
| ENSGALG00000015398 |  | 5.79E-05 | 3.030820016 |
| ENSGALG00000015441 | CD247 | 1.35E-04 | 1.642009479 |
| ENSGALG00000015474 | CD80 | 0.002811834 | 0.990115543 |
| ENSGALG00000015549 | GRPEL1 | 0.041064043 | -0.335186578 |
| ENSGALG00000015645 | GRK4 | 6.02E-04 | 0.870082218 |
| ENSGALG00000015842 | TIAM1 | 4.49E-04 | 1.231561483 |
| ENSGALG00000015902 | CD8B | 0.001256897 | 2.535473993 |
| ENSGALG00000016022 | RUNX1 | 4.49E-04 | 1.457575356 |
| ENSGALG00000016173 | UBASH3A | 9.87E-04 | 1.532871549 |
| ENSGALG00000016205 | PDXK | 0.039578443 | -0.387839821 |
| ENSGALG00000016227 | GPR34 | 0.004472906 | 0.867288309 |
| ENSGALG00000016348 | SAT1 | 0.004952949 | 0.761681762 |
| ENSGALG00000016421 | ITGB1BP1 | 0.00568057 | 0.468535664 |
| ENSGALG00000016446 | ATP6V1C2 | 3.35E-04 | 3.073321946 |
| ENSGALG00000016463 | FAM49A | 4.32E-04 | 1.650086644 |
| ENSGALG00000016471 |  | 0.00420664 | 1.877552282 |
| ENSGALG00000016546 | RBBP7 | 0.020270326 | 0.89823603 |
| ENSGALG00000016556 |  | 0.007413659 | 2.325828205 |
| ENSGALG00000016564 | PTK2B | 0.002113388 | 1.258880648 |
| ENSGALG00000016569 | FANCB | 4.51E-04 | 2.821243274 |
| ENSGALG00000016661 | BLK | 0.002308764 | 1.288788956 |
| ENSGALG00000016687 | P2RY8 | 7.79E-04 | 1.830215466 |
| ENSGALG00000016693 | CSF2RA | 0.002952685 | 1.512359895 |
| ENSGALG00000016769 | CHST10 | 0.002056329 | 1.059248124 |
| ENSGALG00000016788 | IL18RAP | 0.029451753 | 1.460371966 |
| ENSGALG00000016852 | TNFSF13B | 1.19E-05 | 2.579219888 |
| ENSGALG00000016986 | LCP1 | 0.001264211 | 1.387773657 |
| ENSGALG00000016988 | RUBCNL | 5.65E-04 | 0.971904569 |
| ENSGALG00000017046 | POSTN | 4.33E-04 | 1.583231551 |
| ENSGALG00000017274 | CD9 | 0.043936325 | 0.534892734 |
| ENSGALG00000017327 | P2RY6 | 0.012811673 | 1.664857284 |
| ENSGALG00000017362 | HAVCR1 | 0.021171005 | 0.646584461 |
| ENSGALG00000017644 | COTL1 | 4.61E-04 | 2.088026687 |
| ENSGALG00000018700 | PLPP7 | 1.86E-04 | 1.083920051 |
| ENSGALG00000018702 |  | 0.017866064 | 0.865788219 |
| ENSGALG00000019147 |  | 0.001940523 | 1.734675014 |
| ENSGALG00000019555 | SERPINB1 | 5.26E-04 | 2.868711272 |
| ENSGALG00000019755 | CD300L-S1 | 0.007261023 | 3.240853511 |
| ENSGALG00000019795 |  | 0.005042041 | 1.811720921 |
| ENSGALG00000020331 |  | 0.008835261 | 1.577603681 |
| ENSGALG00000020895 |  | 2.65E-04 | 2.042467277 |
| ENSGALG00000021079 | RGS19 | 0.008679969 | 1.214897934 |
| ENSGALG00000021143 | RGS18 | 6.35E-04 | 1.363865302 |
| ENSGALG00000021442 | CARMIL2 | 3.09E-04 | 1.995686102 |
| ENSGALG00000021569 | C1QA | 7.91E-04 | 1.056625271 |
| ENSGALG00000021656 | PTPN22 | 1.10E-04 | 1.800074704 |
| ENSGALG00000022718 | FUT4 | 9.84E-04 | 2.492703751 |
| ENSGALG00000022750 | GPR18 | 1.73E-04 | 1.892557283 |
| ENSGALG00000023347 | PTCHD3 | 7.34E-04 | 1.506215589 |
| ENSGALG00000023411 | CD180 | 0.002098186 | 1.226923903 |
| ENSGALG00000023689 | ASS1 | 0.031325928 | 1.479014057 |
| ENSGALG00000023812 |  | 0.003663842 | 1.219800752 |
| ENSGALG00000023909 |  | 0.004985216 | 2.197327049 |
| ENSGALG00000024020 |  | 0.008820166 | 0.932368187 |
| ENSGALG00000024466 | CCL17 | 8.15E-04 | 2.954974151 |
| ENSGALG00000024490 |  | 0.001174919 | 1.77994679 |
| ENSGALG00000025881 |  | 0.002957523 | 1.695182345 |
| ENSGALG00000025946 | MOB3C | 0.001711182 | 0.888810216 |
| ENSGALG00000025948 | SLA2 | 0.003477495 | 1.413378503 |
| ENSGALG00000026038 |  | 0.008796575 | 0.963395665 |
| ENSGALG00000026070 |  | 0.009786417 | 0.820296584 |
| ENSGALG00000026077 | ALDOC | 0.001147335 | 2.437259188 |
| ENSGALG00000026167 | PIK3R5 | 0.001865066 | 1.462274359 |
| ENSGALG00000026192 | ST8SIA4 | 4.92E-04 | 1.696716065 |
| ENSGALG00000026383 | TMSB4X | 5.38E-04 | 1.518747758 |
| ENSGALG00000026422 |  | 0.00306301 | 1.335993043 |
| ENSGALG00000026768 |  | 4.23E-04 | 3.253030104 |
| ENSGALG00000026781 | ALOX5AP | 0.015876169 | 1.571662363 |
| ENSGALG00000026995 | RHOG | 0.019510355 | 1.279582464 |
| ENSGALG00000027165 | RNASE4 | 4.62E-04 | 1.954383729 |
| ENSGALG00000027209 | SPIC | 0.002553184 | 4.019713313 |
| ENSGALG00000027247 | EOMES | 1.51E-05 | 2.746819887 |
| ENSGALG00000027258 | CDC42SE2 | 1.74E-05 | 1.609187668 |
| ENSGALG00000027305 | SKAP1 | 5.45E-04 | 1.850622821 |
| ENSGALG00000027345 | SLC46A2 | 0.025891481 | 1.583274798 |
| ENSGALG00000027365 | HIVEP3 | 0.001374043 | 2.549802307 |
| ENSGALG00000027407 |  | 0.0039745 | 2.172770464 |
| ENSGALG00000027587 |  | 1.41E-04 | 2.640425108 |
| ENSGALG00000027629 | GNG2 | 0.026079461 | 1.038602884 |
| ENSGALG00000027630 | NPR2 | 0.031550475 | 0.97342493 |
| ENSGALG00000027747 | TROJANZ | 0.012491779 | 1.481459647 |
| ENSGALG00000027761 | TNFAIP8 | 4.29E-04 | 1.114629029 |
| ENSGALG00000027777 | CNN2 | 0.002410442 | 1.374006152 |
| ENSGALG00000027961 | PCASP2 | 0.00456777 | 1.259454354 |
| ENSGALG00000028016 |  | 0.010693162 | 1.889996579 |
| ENSGALG00000028256 | CCL19 | 6.55E-04 | 2.425692994 |
| ENSGALG00000028318 | CDKN1A | 2.76E-04 | 2.896352663 |
| ENSGALG00000028341 |  | 0.005143071 | 2.039379107 |
| ENSGALG00000028448 | SH2D1B | 0.002280009 | 1.939747721 |
| ENSGALG00000028536 | MPEG1 | 0.006546441 | 1.136865902 |
| ENSGALG00000028790 | DNASE2B | 4.03E-04 | 1.031654404 |
| ENSGALG00000028803 | B3GNT4 | 6.35E-04 | 1.502002818 |
| ENSGALG00000029027 | Sep-12 | 0.011414663 | 1.199482936 |
| ENSGALG00000029077 | CBL | 0.003144181 | 0.874405713 |
| ENSGALG00000029244 | SMAP2 | 0.002896505 | 0.856695931 |
| ENSGALG00000029270 | GATA3 | 0.004577763 | 1.236184891 |
| ENSGALG00000029354 | GMIP | 9.47E-04 | 1.486032516 |
| ENSGALG00000029381 | TAP2 | 0.004138318 | 1.279343424 |
| ENSGALG00000029395 | GSTM3 | 0.005196956 | -0.841447046 |
| ENSGALG00000029502 | CLIC2 | 0.00886239 | 1.17123571 |
| ENSGALG00000029520 |  | 0.024037195 | 1.368996691 |
| ENSGALG00000029552 |  | 0.026581462 | 1.551838366 |
| ENSGALG00000029569 |  | 0.002791156 | 1.722141085 |
| ENSGALG00000029669 | C1QC | 7.30E-05 | 1.202216027 |
| ENSGALG00000029718 |  | 0.002656645 | 1.722276895 |
| ENSGALG00000029724 | MTURN | 0.00166308 | -0.55164536 |
| ENSGALG00000029971 | PGLYRP2 | 0.025765036 | -0.676431195 |
| ENSGALG00000030179 | VSIR | 0.023675637 | 1.011543102 |
| ENSGALG00000030432 | RASSF2 | 0.001865066 | 1.34685204 |
| ENSGALG00000030436 | ND3 | 0.005271889 | -0.766995759 |
| ENSGALG00000030602 | ADAM33 | 0.014924767 | 1.045515147 |
| ENSGALG00000030907 | CSF3 | 0.002973736 | 2.532798823 |
| ENSGALG00000030940 | BLB2 | 0.001223862 | 1.962959659 |
| ENSGALG00000031000 | APRT | 0.002126711 | 1.403243911 |
| ENSGALG00000031149 |  | 0.047611235 | 1.227876598 |
| ENSGALG00000031276 |  | 0.047344835 | -0.483809069 |
| ENSGALG00000031593 | TMSB15B | 0.001794569 | 1.468937509 |
| ENSGALG00000031694 |  | 1.41E-04 | 2.502959006 |
| ENSGALG00000031709 |  | 0.049555253 | 1.256527688 |
| ENSGALG00000031735 | CD5 | 0.026380974 | 1.458063308 |
| ENSGALG00000031794 | PDCD1LG2 | 5.21E-04 | 1.883994269 |
| ENSGALG00000031835 | TMEM68 | 0.005699462 | 0.901746548 |
| ENSGALG00000031862 | BCL11B | 0.001733185 | 1.653009682 |
| ENSGALG00000031978 | TARP | 6.67E-06 | 1.970062884 |
| ENSGALG00000032155 |  | 6.54E-04 | 1.363952476 |
| ENSGALG00000032190 | MICAL1 | 2.02E-04 | 1.464254208 |
| ENSGALG00000032260 | IL10RA | 5.31E-04 | 2.211908903 |
| ENSGALG00000032340 |  | 0.001678007 | 1.15855679 |
| ENSGALG00000032456 | COII | 0.005409319 | -0.7904391 |
| ENSGALG00000032465 | ATP8 | 0.010873314 | -0.658499737 |
| ENSGALG00000032469 | ARHGAP45 | 8.16E-04 | 1.257942058 |
| ENSGALG00000032526 | B3GNT5 | 1.71E-05 | 3.716005089 |
| ENSGALG00000032530 | SASH3 | 0.003397645 | 1.004536007 |
| ENSGALG00000032588 | ARPC1B | 6.37E-04 | 1.751197984 |
| ENSGALG00000032626 |  | 2.77E-04 | 1.891540511 |
| ENSGALG00000032658 |  | 8.97E-04 | 1.352199874 |
| ENSGALG00000032677 |  | 0.004642076 | 1.119019502 |
| ENSGALG00000032701 | LY96 | 8.42E-04 | 2.027903055 |
| ENSGALG00000033094 |  | 0.001567211 | 2.335212416 |
| ENSGALG00000033226 | FAM107B | 5.77E-04 | 1.333038444 |
| ENSGALG00000033234 | JAK3 | 0.001301576 | 1.719322876 |
| ENSGALG00000033278 | NLRC3 | 9.74E-04 | 1.514297706 |
| ENSGALG00000033466 |  | 2.10E-05 | 3.527100187 |
| ENSGALG00000033507 | PLCG2 | 4.61E-04 | 1.396412777 |
| ENSGALG00000033520 | DCSTAMP | 4.06E-04 | 4.459858483 |
| ENSGALG00000033618 |  | 0.006532781 | 0.817582908 |
| ENSGALG00000033635 | PTGS2 | 0.021062661 | 0.979371426 |
| ENSGALG00000033885 | RAP1GAP2 | 0.005494821 | 1.143446838 |
| ENSGALG00000033898 |  | 6.15E-04 | 1.685172146 |
| ENSGALG00000033932 | BF1 | 0.006331549 | 1.076345 |
| ENSGALG00000034021 |  | 2.30E-05 | 2.257047657 |
| ENSGALG00000034085 |  | 0.006562565 | 1.25335119 |
| ENSGALG00000034155 |  | 0.045219986 | 0.637913666 |
| ENSGALG00000034251 | SLC9A1 | 0.009400957 | 0.97295649 |
| ENSGALG00000034349 |  | 0.009492654 | 1.595688353 |
| ENSGALG00000034478 | CCL4 | 0.001945538 | 3.701639941 |
| ENSGALG00000034556 |  | 0.034251831 | 1.119033476 |
| ENSGALG00000034590 | CELF2 | 2.32E-04 | 1.621911135 |
| ENSGALG00000034855 | HK2 | 0.003606714 | 1.244032485 |
| ENSGALG00000034950 | SMPD3 | 0.007783737 | 1.26351977 |
| ENSGALG00000034964 | SYTL1 | 1.41E-04 | 1.991592709 |
| ENSGALG00000034970 | PRKCQ | 6.52E-05 | 1.877956074 |
| ENSGALG00000035007 | USP35 | 0.038645115 | 1.110325575 |
| ENSGALG00000035075 | TAP1 | 0.003652847 | 1.867189466 |
| ENSGALG00000035194 | ARHGAP25 | 0.003812365 | 1.220560652 |
| ENSGALG00000035325 | PMAIP1 | 0.010580653 | 1.813913396 |
| ENSGALG00000035334 | COX3 | 0.004503688 | -0.680992421 |
| ENSGALG00000035386 | NCKAP1L | 0.005042041 | 1.213347583 |
| ENSGALG00000035473 |  | 1.10E-04 | 1.612921296 |
| ENSGALG00000035498 | INPP5D | 3.68E-04 | 1.244960207 |
| ENSGALG00000035505 | AOAH | 8.64E-04 | 1.737960394 |
| ENSGALG00000035589 |  | 0.001594855 | 1.97230272 |
| ENSGALG00000035718 | DOK1 | 0.008047806 | 1.152606995 |
| ENSGALG00000035726 | FLT3 | 0.024966017 | 0.924226471 |
| ENSGALG00000035733 | CCR7 | 0.001794569 | 1.647312097 |
| ENSGALG00000035809 |  | 0.048257784 | 1.120713185 |
| ENSGALG00000035825 |  | 0.003017057 | 2.509032649 |
| ENSGALG00000035856 | CD48 | 4.45E-04 | 1.926076627 |
| ENSGALG00000036093 | PTPN7 | 1.83E-05 | 1.93902374 |
| ENSGALG00000036395 |  | 2.76E-06 | 2.249301921 |
| ENSGALG00000036470 | P2RY12 | 0.012353283 | 1.580373117 |
| ENSGALG00000036547 |  | 6.17E-04 | 1.193456132 |
| ENSGALG00000036645 | KIF15 | 5.21E-04 | 2.538715763 |
| ENSGALG00000036942 | KCNA3 | 2.65E-04 | 2.969202783 |
| ENSGALG00000037559 |  | 0.012181597 | 2.359833147 |
| ENSGALG00000037645 |  | 0.016798617 | 1.625061553 |
| ENSGALG00000037675 | COL14A1 | 0.002422976 | -1.024136529 |
| ENSGALG00000037929 | BIN2 | 0.001411896 | 1.546803637 |
| ENSGALG00000037943 | PRKCB | 4.39E-04 | 1.604601987 |
| ENSGALG00000038069 | CCR8 | 0.001746994 | 1.879040539 |
| ENSGALG00000038096 | NOS2 | 0.007721661 | 3.282019851 |
| ENSGALG00000038110 | DGKA | 0.006331549 | 0.802268172 |
| ENSGALG00000038136 |  | 0.001384911 | 1.265216189 |
| ENSGALG00000038146 |  | 0.006062658 | 1.096206141 |
| ENSGALG00000038217 | CD82 | 0.013267603 | 0.702676777 |
| ENSGALG00000038256 |  | 0.045254599 | 1.207061899 |
| ENSGALG00000038298 |  | 0.002488535 | 1.10380801 |
| ENSGALG00000038374 | SNX20 | 5.77E-04 | 1.013689147 |
| ENSGALG00000038393 | DMB2 | 0.008173841 | 1.532490172 |
| ENSGALG00000038411 | CENPL | 0.008173841 | 2.351743901 |
| ENSGALG00000038504 | MID1 | 0.026271526 | -0.901609493 |
| ENSGALG00000038536 | SH3BGRL3 | 0.007609062 | 1.021086283 |
| ENSGALG00000038559 | CD8A | 0.003196932 | 1.961370312 |
| ENSGALG00000038636 | LAPTM5 | 0.001945538 | 1.303612997 |
| ENSGALG00000038671 |  | 0.008613885 | 1.059123341 |
| ENSGALG00000038902 | PIM1 | 9.92E-04 | 1.651705987 |
| ENSGALG00000038918 |  | 0.001471021 | 3.870695237 |
| ENSGALG00000038943 | RASSF5 | 0.005699462 | 1.114847459 |
| ENSGALG00000039054 | AT1 | 0.002811834 | 2.077291183 |
| ENSGALG00000039084 | EVI2A | 5.14E-05 | 2.7946635 |
| ENSGALG00000039156 |  | 0.016072987 | 1.309981783 |
| ENSGALG00000039200 | AXIN2 | 0.001489646 | -0.786714709 |
| ENSGALG00000039221 |  | 1.41E-04 | 1.618686694 |
| ENSGALG00000039264 | SLA | 1.41E-04 | 1.892546762 |
| ENSGALG00000039432 | P2RY13 | 0.018294741 | 1.223922004 |
| ENSGALG00000039461 | TNFRSF1A | 0.047155345 | 0.551120362 |
| ENSGALG00000039609 |  | 0.004210727 | 1.411665784 |
| ENSGALG00000039647 |  | 4.82E-05 | 5.833335662 |
| ENSGALG00000039682 | HCLS1 | 0.001007586 | 1.622481759 |
| ENSGALG00000039705 | GFPT2 | 0.020022376 | 0.793048202 |
| ENSGALG00000039966 |  | 0.001555511 | 1.671214923 |
| ENSGALG00000039982 | IRF5 | 0.003987124 | 1.398972546 |
| ENSGALG00000040002 |  | 1.83E-05 | 2.680605248 |
| ENSGALG00000040136 | PSTPIP2 | 0.015365314 | 1.462644001 |
| ENSGALG00000040162 |  | 2.33E-04 | 3.292725117 |
| ENSGALG00000040275 | RCSD1 | 0.001008126 | 1.302268411 |
| ENSGALG00000040351 |  | 6.35E-04 | 1.761297759 |
| ENSGALG00000040418 | DGAT2 | 1.92E-05 | 1.290437082 |
| ENSGALG00000040549 | HTR7 | 0.005272256 | 1.526656982 |
| ENSGALG00000040561 | GPR171 | 1.01E-05 | 3.056873421 |
| ENSGALG00000040621 | IL18R1 | 0.003148756 | 1.591169135 |
| ENSGALG00000040926 | SAMSN1 | 2.28E-04 | 1.694882699 |
| ENSGALG00000041034 | MIA3 | 0.041795979 | -0.34026744 |
| ENSGALG00000041091 | ATP6 | 0.006084163 | -0.720015559 |
| ENSGALG00000041140 | HTRA1 | 0.001912239 | 1.174055669 |
| ENSGALG00000041149 |  | 0.002283748 | 1.570042194 |
| ENSGALG00000041202 | FBXO32 | 0.013497209 | 0.551754242 |
| ENSGALG00000041298 |  | 0.001567211 | 1.678752323 |
| ENSGALG00000041380 | BF2 | 0.045644381 | 1.018285968 |
| ENSGALG00000041577 | ITGA4 | 7.71E-05 | 1.974965157 |
| ENSGALG00000041611 |  | 0.004299829 | 1.640598021 |
| ENSGALG00000041631 | C3orf70 | 0.020979475 | 1.074328097 |
| ENSGALG00000041635 | CAMK2D | 0.002005159 | 1.530463438 |
| ENSGALG00000041787 | PLA2G15 | 4.79E-04 | 0.89864722 |
| ENSGALG00000042059 |  | 0.019135764 | 5.127805372 |
| ENSGALG00000042101 |  | 0.004913662 | 1.072964446 |
| ENSGALG00000042227 | GNLY | 8.04E-05 | 2.56694168 |
| ENSGALG00000042351 | PIK3R6 | 0.003477495 | 1.52109561 |
| ENSGALG00000042458 | ACTN1 | 0.001037119 | 1.208390847 |
| ENSGALG00000042471 | IL2RA | 0.021902641 | 1.161444593 |
| ENSGALG00000042534 | NFATC1 | 0.005789539 | 1.564559063 |
| ENSGALG00000042587 |  | 0.006106972 | 1.431172937 |
| ENSGALG00000042739 | TACC1 | 0.003059409 | 1.154938179 |
| ENSGALG00000043051 | CLU | 0.006819281 | 1.595199819 |
| ENSGALG00000043052 | CD1B | 0.007575586 | 0.999917729 |
| ENSGALG00000043302 | LAT2 | 5.20E-04 | 2.204451939 |
| ENSGALG00000043372 | RUNX3 | 2.32E-04 | 1.859769252 |
| ENSGALG00000043484 |  | 0.033110614 | 1.061017518 |
| ENSGALG00000043487 |  | 0.001481719 | 1.625129038 |
| ENSGALG00000043582 |  | 3.57E-05 | 7.743527665 |
| ENSGALG00000043603 | CCL5 | 0.00399956 | 2.345394958 |
| ENSGALG00000043650 | GFI1 | 0.00144412 | 1.800324642 |
| ENSGALG00000043688 |  | 0.00154712 | 0.928999016 |
| ENSGALG00000043734 | LIPG | 0.006819281 | 2.099903177 |
| ENSGALG00000043768 | ND2 | 0.013197269 | -0.778950336 |
| ENSGALG00000043817 |  | 0.003254773 | 1.923807606 |
| ENSGALG00000043877 |  | 0.045254599 | 0.469307014 |
| ENSGALG00000044135 |  | 0.025543039 | 2.243249595 |
| ENSGALG00000044175 |  | 5.98E-06 | 2.582471071 |
| ENSGALG00000044229 |  | 0.04778586 | 1.459781292 |
| ENSGALG00000044230 |  | 8.64E-04 | 1.216125494 |
| ENSGALG00000044319 |  | 4.61E-04 | 1.414376795 |
| ENSGALG00000044339 |  | 0.010173355 | 1.154692623 |
| ENSGALG00000044353 |  | 2.51E-04 | 7.761815193 |
| ENSGALG00000044442 |  | 0.003477495 | 1.19543979 |
| ENSGALG00000044449 |  | 2.57E-04 | 4.687902479 |
| ENSGALG00000044505 | SH2D2A | 0.047344835 | 1.200890339 |
| ENSGALG00000044586 |  | 0.010882423 | 2.204170322 |
| ENSGALG00000044720 |  | 0.006702447 | 3.752414361 |
| ENSGALG00000044763 | GPR82 | 0.039578443 | 1.1748205 |
| ENSGALG00000045021 |  | 0.004264202 | 1.747629578 |
| ENSGALG00000045034 |  | 0.012555358 | 0.77583364 |
| ENSGALG00000045053 |  | 0.00144412 | 2.45277912 |
| ENSGALG00000045115 |  | 0.006654619 | 0.646302121 |
| ENSGALG00000045122 | AKR1B10L1 | 0.001041129 | 1.135608457 |
| ENSGALG00000045173 |  | 0.012576951 | 1.270156307 |
| ENSGALG00000045266 |  | 0.010541448 | 2.284373254 |
| ENSGALG00000045392 |  | 5.44E-06 | 8.81692762 |
| ENSGALG00000045515 |  | 0.005902139 | 2.158970541 |
| ENSGALG00000045545 |  | 0.023136509 | 2.897238411 |
| ENSGALG00000045581 |  | 0.001965922 | 1.511832118 |
| ENSGALG00000045607 | CXCL13 | 8.88E-04 | 5.148791006 |
| ENSGALG00000045879 |  | 6.54E-05 | 9.157157109 |
| ENSGALG00000045916 |  | 3.09E-04 | 2.883941108 |
| ENSGALG00000045925 |  | 9.22E-04 | 1.81353448 |
| ENSGALG00000046032 | CD83 | 0.007895467 | 1.295144218 |
| ENSGALG00000046130 |  | 8.56E-04 | 2.006189431 |
| ENSGALG00000046192 |  | 3.57E-05 | 3.030207547 |
| ENSGALG00000046222 |  | 0.002269333 | 1.52067346 |
| ENSGALG00000046379 |  | 0.049104694 | 1.331924994 |
| ENSGALG00000046532 | C15orf48 | 4.24E-04 | 2.954597052 |
| ENSGALG00000046650 |  | 0.003683887 | 1.507691717 |
